# NuRD mediates mitochondrial stress–induced longevity via chromatin remodeling in response to acetyl-CoA level

**DOI:** 10.1126/sciadv.abb2529

**Published:** 2020-07-31

**Authors:** Di Zhu, Xueying Wu, Jun Zhou, Xinyu Li, Xiahe Huang, Jiasheng Li, Junbo Wu, Qian Bian, Yingchun Wang, Ye Tian

**Affiliations:** 1 State Key Laboratory of Molecular Developmental Biology, Institute of Genetics and Developmental Biology, Chinese Academy of Sciences, Beijing 100101, China.; 2 University of Chinese Academy of Sciences, Beijing 100093, China.; 3 Shanghai Institute of Precision Medicine, Ninth People's Hospital, Shanghai Jiao Tong University School of Medicine, Shanghai 200125, China.; 4 Center for Excellence in Animal Evolution and Genetics, Chinese Academy of Sciences, Kunming 650223, China.

## Abstract

Mild mitochondrial stress experienced early in life can have beneficial effects on the life span of organisms through epigenetic regulations. Here, we report that acetyl–coenzyme A (CoA) represents a critical mitochondrial signal to regulate aging through the chromatin remodeling and histone deacetylase complex (NuRD) in *Caenorhabditis elegans*. Upon mitochondrial stress, the impaired tricarboxylic acid cycle results in a decreased level of citrate, which accounts for reduced production of acetyl-CoA and consequently induces nuclear accumulation of the NuRD and a homeodomain-containing transcription factor DVE-1, thereby enabling decreased histone acetylation and chromatin reorganization. The metabolic stress response is thus established during early life and propagated into adulthood to allow transcriptional regulation for life-span extension. Furthermore, adding nutrients to restore acetyl-CoA production is sufficient to counteract the chromatin changes and diminish the longevity upon mitochondrial stress. Our findings uncover the molecular mechanism of the metabolite-mediated epigenome for the regulation of organismal aging.

## Teaser

Acetyl-CoA links mitochondrial stress to the nuclear epigenome via NuRD complex for life-span regulation.

## INTRODUCTION

Metabolic homeostasis and aging are intimately linked (*1*). The cellular processes that respond to metabolic stress will influence the bioenergetic status of the cells, thus affecting the fitness of the entire organism. Metabolic stress in early life appears to be capable of restructuring chromatin, leaving a durable epigenetic change that may even influence the aging process (*2*).

A regulatory center for these epigenetic changes lies in the mitochondria (*3*). In addition to generating the bulk of adenosine 5′-triphosphate (ATP) through the tricarboxylic acid (TCA) cycle and oxidative phosphorylation (OXPHOS) to maintain cellular homeostasis, mitochondria also participate in biosynthesis of molecules such as lipids, heme, iron-sulfur clusters, and intermediate metabolites that can signal to the rest of the cell, making mitochondria central to diverse biological processes (*4*). Therefore, continuous communication between mitochondria and the nucleus allows cells and organisms to integrate nutrient availability and energy demand to ensure metabolic homeostasis (*5*).

Although complete or near-complete loss of mitochondrial function is detrimental, partial suppression of the mitochondrial activity has been shown to promote longevity in worms, flies, and mice (*6*–*9*). Work in *Caenorhabditis elegans* has shown that decreasing mitochondrial electron transport chain (ETC) activity during early life induces extensive chromatin restructuring that is essential for activation of the mitochondrial unfolded protein response (UPR^mt^), a process that promotes the recovery of mitochondrial protein homeostasis and stress-induced longevity (*10*, *11*). Specifically, mitochondrial stress–induced global chromatin compaction is manifested by histone H3K9 methyltransferase SETDB1/MET-2 and its cofactor ATF7IP/LIN-65 to avoid transcription of nonessential genes under stress conditions, while the activation of the stress-related UPR^mt^ genes is mediated by the histone H3K27 demethylases JMJD-1.2 and JMJD-3.1, thus eliciting a persistent response resulting in life-span extension (*11*, *12*).

UPR^mt^ is a transcriptional response to induce expression of mitochondrial chaperones and proteases to restore protein homeostasis within mitochondria (*13*). Moreover, UPR^mt^ also promotes the rewiring of cellular metabolism to relieve mitochondrial stress and promote survival (*14*). In *C. elegans*, UPR^mt^ is induced when mitochondrial protein homeostasis is disrupted, leading to a decreased import efficiency, thus allowing the transcription factor (TF) ATFS-1 to enter the nucleus instead of being imported to the mitochondria where it is quickly degraded (*15*). Another TF and chromatin remodeling factor DVE-1 also participate in the UPR^mt^ signaling, but unlike ATFS-1, its nuclear translocation is not dependent on the mitochondrial import efficiency (*16*). The signal generated from stressed mitochondria that regulates DVE-1 nuclear translocation and induces chromatin reorganization remains unknown.

It is increasingly recognized that the metabolic status of a cell exerts a profound and dynamic influence on global chromatin modifications since many metabolic intermediates [e.g., folate, acetyl–coenzyme A (acetyl-CoA), and α-ketoglutarate (α-KG)] act as substrates for enzymes that modify signaling proteins, metabolic enzymes, and chromatin proteins (*17*). Mitochondria are the key suppliers of metabolites for epigenetic regulation. Acetyl-CoA is a substrate for histone and protein acetylation (*18*, *19*), while levels of methyl donor *S*-adenosylmethionine can influence histone and DNA methylation (*20*). In addition, α-KG generated in the TCA cycle in the mitochondria serves as an essential cofactor of the histone demethylases and DNA methylases; conversely, succinate and fumarate inhibit these α-KG–dependent enzymes (*21*). Thus, mitochondrial perturbations could alter the nuclear epigenome through various mitochondrial-derived metabolites to regulate cell proliferation and the aging process.

Here, we studied the homeodomain-containing TF DVE-1 that is essential for the UPR^mt^ and identified the nucleosome remodeling and histone deacetylase (NuRD) complex that mediate DVE-1 subcellular localization in response to mitochondrial stress. We further found that the decreased level of mitochondrial-derived acetyl-CoA upon perturbations of mitochondrial function decreased histone acetylation levels, enabling global chromatin reorganization manifested by the NuRD complex. Restoration of the acetyl-CoA level by providing substrates and nutrients required for acetyl-CoA production prevented the decrease of histone acetylation and inhibited the chromatin reorganization under mitochondrial stress conditions. Collectively, our results revealed that acetyl-CoA is the signal for the dysfunctional mitochondria that regulates organismal aging through NuRD-mediated chromatin remodeling.

## RESULTS

### The NuRD complex is required for UPR^mt^ signaling

**Fig. 1:**
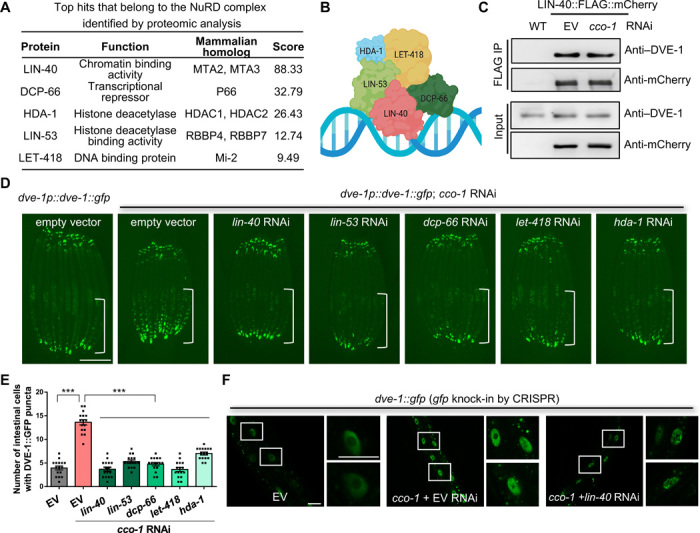
Identification and characterization of DVE-1–associated NuRD complex in UPR^mt^ signaling. (**A**) List of DVE-1–interacting proteins that belong to the NuRD complex identified by IP-MS experiments. *Dve-1p::dve-1::gfp* transgenic worms grown on empty vector (EV) and *cco-1* RNAi bacteria were used for anti-GFP IP and MS. All proteins that belong to the NuRD complex were detected multiple times in three biological replicates. Score reflects the combined scores of all observed mass spectra that can be matched to amino acid sequences within a protein. A higher score indicates a more confident match. (**B**) Simplified model of NuRD complex subunits in *C. elegans*. (**C**) Validation of the interaction between DVE-1 and the NuRD complex. *Lin-40p::lin-40::Flag::mCherry* transgenic animals grown on EV or *cco-1* RNAi bacteria were used for in vivo IP experiments followed by Western blot analyses using anti–DVE-1 antibody. Wild-type (WT) animals were used as negative control. (**D**) Representative photomicrographs of *dve-1p::dve-1::gfp* reporter in day-1 adult animals grown on EV, *cco-1* + EV, *cco-1* + *lin-40*, *cco-1* + *lin-53*, *cco-1* + *dcp-66*, *cco-1* + *let-418*, or *cco-1* + *hda-1* double-RNAi bacteria from hatch. The posterior region of the intestine where DVE-1::GFP is induced or suppressed is highlighted with a white line. Scale bar, 250 μm. (**E**) Quantification of the number of intestinal nuclei with GFP signal per worm. The treatment is as shown in (D). ***P < 0.0001 via *t* test. Error bars, SEM; *n* ≥ 15 worms. (**F**) Representative photomicrographs of *dve-1::gfp* animals grown on EV, *cco-1* + EV, or *cco-1 + lin-40* double-RNAi bacteria from hatch. Dash rectangles highlight the areas enlarged and shown on the right. Scale bars, 25 μm.

Previous work has established that mitochondrial perturbations induce nuclear redistribution of DVE-1, a TF that is essential for the UPR^mt^ in *C. elegans (**11*, *16*). To search for the DVE-1 interacting proteins, we performed DVE-1::GFP (green fluorescent protein) immunoprecipitation (IP) of control and *cco-1* (cytochrome C oxidase-1; a subunit of complex IV in the ETC) RNA interference (RNAi)–treated animals and used mass spectrometry (MS) to identify proteins that potentially interact with DVE-1. The MS analysis revealed that the NuRD complex interacts with DVE-1 under both normal and mitochondrial stress conditions (Fig. 1A). The NuRD complex is a highly conserved regulator for chromatin structure and transcription and contains ATP-dependent remodeling enzyme Mi-2/LET-418, histone deacetylase 1/HDA-1, and other components including MTA/LIN-40, RBBP/LIN-53, and P66/DCP-66 (Fig. 1B) (*22*). The subunits of the NuRD complex were all identified in the DVE-1::GFP IP-MS analysis (see data file S1 for the IP-MS list).

To confirm this coimmunoprecipitation result, we generated transgenic animals expressing *lin-40p::lin-40::Flag::mCherry*, the scaffolds for NuRD complex assembly, and also developed an antibody against DVE-1 in *C. elegans* (fig. S1A). The reciprocal LIN-40::FLAG::mCherry pulldowns also identified DVE-1 as an interacting partner for the NuRD complex under both normal and mitochondrial stress conditions (Fig. 1C). In addition, we undertook an unbiased ethylmethane sulfonate mutagenesis screen to identify genes that are essential for nuclear accumulation of DVE-1::GFP in the intestine of animals in response to the neuronal mitochondrial stress, the cell-nonautonomous UPR^mt^ model that we established in our previous study (*23*). The induction of the UPR^mt^ in the neurons can be communicated to peripheral tissues (e.g., intestine) that have not experienced the stress, preparing the entire organism to cope with local mitochondrial perturbations (*10*). Coincidently, one of the mutants, *lin-40*(*yth27*, G612E), showed a strong suppression of the DVE-1::GFP signal in the intestinal nuclei in response to both neuronal mitochondrial stresses and *cco-1* RNAi treatment. Furthermore, the suppression phenotype in *lin-40*(*yth27*) mutants can be rescued by the transgene *lin-40p::lin-40::Flag::mCherry* (fig. S1, B and C). Likewise, RNAi against other components of the NuRD complex such as *lin-53*, *dcp-66*, and *let-418* and histone deacetylase *hda-1* also showed similar suppression of DVE-1::GFP signal in the intestine upon *cco-1* RNAi treatment (Fig. 1, D and E). The role of the NuRD complex in the regulation of DVE-1 nuclear localization upon mitochondrial stress was further verified in animals with CRISPR-Cas9–mediated GFP knock-in at the locus of endogenous *dve-1* C terminus (*dve-1::gfp*). *lin-40* mutants showed a lower level of *dve-1::gfp* signal in the intestinal nuclei compared to wild-type (WT) control animals under *cco-1* RNAi conditions (Fig. 1F). In addition, suppression of the DVE-1::GFP signal was not due to the down-regulation of *dve-1* transcription in the absence of the NuRD complex subunits (fig. S1, D to H).

To test whether the NuRD complex was specifically required for the mitochondrial stress response, we examined the involvement of the NuRD complex in either the endoplasmic reticulum (ER) or cytoplasmic UPR response. To this end, we fed *lin-40* RNAi bacteria to animals harboring a reporter (*hsp-4p::gfp*) for the UPR^ER^ (*24*). Animals were then treated with tunicamycin, an ER-specific stressor that blocks N-linked glycosylation and induces UPR^ER^ (*25*). *lin-40* did not affect the up-regulation of the *hsp-4p::gfp* reporter by tunicamycin (fig. S1I). Similarly, we observed that animals grown on *lin-40* RNAi bacteria were fully capable of eliciting a heat shock response, as measured by the *hsp-16.2p::gfp* reporter (fig. S1J) (*26*). Collectively, these results indicate that the NuRD complex plays a specific role in regulating the mitochondrial stress response.

### NuRD complex subunits accumulate in the nucleus in response to the mitochondrial stress

To understand how the NuRD complex is regulated under mitochondrial stress conditions, we examined the expression and the localization of the NuRD complex components upon *cco-1* RNAi treatment. The expression level of the *lin-53p::gfp* reporter was slightly up-regulated upon *cco-1* RNAi treatment in the intestine in which tissue UPR^mt^ induction typically appears strongest upon mitochondrial perturbation (fig. S2A). The endogenous mRNA levels of other NuRD subunits, including *lin-40*, *hda-1*, and *let-418*, were also up-regulated upon *cco-1* RNAi treatment (fig. S2B).

**Fig. 2:**
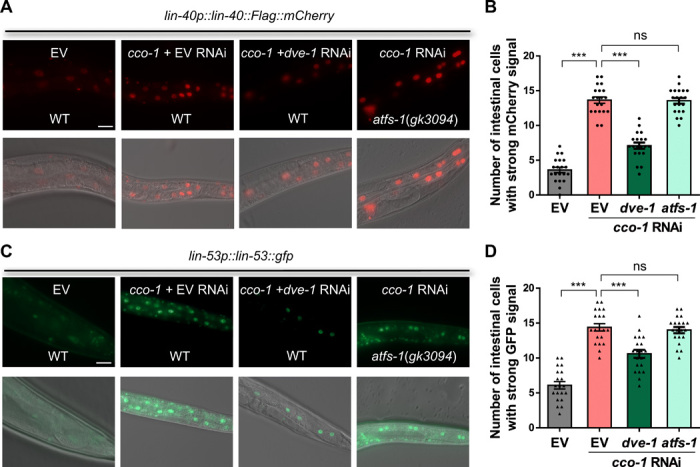
NuRD complex subunits accumulate in the nucleus in response to mitochondrial stress. (**A**) Representative photomicrographs of LIN-40::mCherry animals in a WT or *atfs-1* mutant background grown on EV, *cco-1* + EV, or *cco-1 + dve-1* double-RNAi bacteria from hatch. Scale bar, 25 μm. (**B**) Quantification of the number of intestinal nuclei with strong mCherry signal in animals as shown in (A). (**C**) Representative photomicrographs of *lin-53p::lin-53::gfp* animals in a WT or *atfs-1* mutant background grown on EV, *cco-1* + EV, or *cco-1 + dve-1* double-RNAi bacteria from hatch. Scale bar, 25 μm. (**D**) Quantification of the number of intestinal nuclei with strong GFP signal in animals as shown in (C). ***P < 0.0001, ns denotes *P* > 0.05 via *t* test. Error bars, SEM; *n* ≥ 15 worms.

Next, to determine whether the NuRD complex is regulated posttranslationally, we created transgenic animals expressing the subunits of the NuRD complex, *lin-40p::lin-40::Flag::mCherry* and *lin-53p::lin-53::gfp*. Similar to the behavior of the DVE-1::GFP, both LIN-40::Flag::mCherry and LIN-53::GFP fluorescent signals became stronger and accumulated in the nuclei of the intestine upon *cco-1* RNAi treatment (Fig. 2, A to D). Western blot analyses against mCherry expression indicated that LIN-40::mCherry protein levels accumulated after prolonged mitochondrial stress (fig. S2, C and D). Likewise, the animals with the CRISPR-Cas9–mediated GFP knock-in at the endogenous *lin-40* C terminus (*lin-40::gfp*) also showed that the LIN-40 accumulated in the intestinal nuclei upon mitochondrial stress, while normally, the LIN-40::GFP signal became weaker during the aging process (fig. S2E) (see Discussion). Moreover, nuclear accumulation of the NuRD subunits LIN-40 and LIN-53 upon *cco-1* RNAi treatment was partially suppressed in animals with *dve-1* RNAi treatment, but not suppressed in *atfs-1* mutants (Fig. 2, A to D). Collectively, these results suggest that mitochondrial stress induces nuclear accumulation of the NuRD complex.

### The NuRD complex is required for the mitochondrial stress–induced global chromatin reorganization

**Fig. 3:**
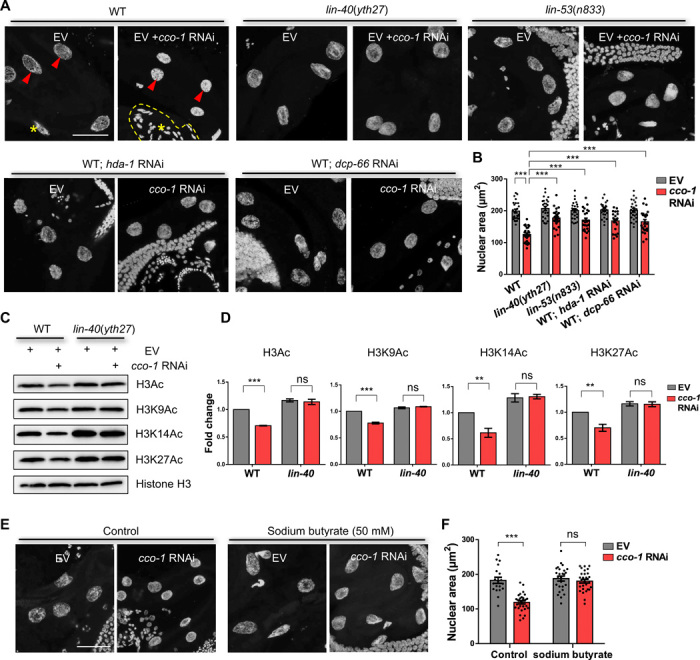
The NuRD complex is required for the mitochondrial stress–induced global chromatin reorganization. (**A**) Representative maximal intensity projection images of 4′,6-diamidino-2-phenylindole (DAPI) immunostaining of intestinal nuclei in day-1 adult WT, *lin-40*, or *lin-53* mutant animals grown on EV, *cco-1* + EV, EV + *hda-1*, *cco-1 + hda-1*, EV *+ dcp-66*, or *cco-1* + *dcp-66* double-RNAi bacteria from hatch. The red arrow indicates the intestinal nucleus; the area circled by a yellow dotted line or asterisk denotes nucleus of other tissues (e.g., germ cell and muscle). DAPI (grey); Scale bar, 25 μm. (**B**) Quantification of the intestinal nuclear maximum cross-section area at day 1 of adulthood in animals as shown in (A). *n* ≥ 25 nuclei. (**C**) Immunoblots of histone H3Ac, H3K9Ac, H3K14Ac, and H3K27Ac in WT or *lin-40* animals grown on EV or *cco-1* RNAi. Anti-histone H3 serves as loading control. (**D**) Quantified mean data of histone H3Ac, H3K9Ac, H3K14Ac, and H3K27Ac levels (relative to histone H3). *n* = 3. (**E**) Representative maximal intensity projection images of DAPI immunostaining of intestinal nuclei at day 1 of adulthood in animals grown on EV or *cco-1* RNAi from hatch with or without sodium butyrate treatment. DAPI (grey); Scale bar, 25 μm. (**F**) Quantification of the intestinal nuclear maximum cross-section area at day 1 of adulthood in animals, as shown in (E). *n* ≥ 25 nuclei. **P < 0.01 and ***P < 0.0001 and ns denotes *P* > 0.05 via *t* test. Error bars, SEM.

Previously, we showed that mitochondrial stress induced global chromatin reorganization of the intestinal nuclei in *C. elegans*. The intestinal nuclei appeared smaller and more condensed in animals with *cco-1* RNAi treatment than those in unstressed animals (Fig. 3, A and B) (*11*). Given the role of the NuRD complex in chromatin remodeling, we hypothesized that the NuRD complex might contribute to the changes in chromatin structure upon mitochondrial stress. We thus analyzed the status of the chromatin structure in both *lin-40*(*yth27*) and *lin-53*(*n833*) mutant animals and in animals after RNA-mediated knockdown of the other NuRD subunits, with or without mitochondrial stress (*cco-1* RNAi). Under normal conditions, loss of NuRD components did not significantly change the chromatin structure when compared to WT animals. Under mitochondrial stress conditions, however, a substantial fraction of nuclei in animals in the absence of the NuRD components remained enlarged in size and exhibited relatively loose structure when compared to WT animals, while the lengths of the worms are not significantly different between WT and NuRD mutants upon *cco-1* RNAi treatment (Fig. 3, A and B, and fig. S3A). These results indicate that the global chromatin changes upon *cco-1* RNAi treatment were strongly dependent on the NuRD complex.

Given the role of the NuRD complex in histone deacetylation, we further examined the histone acetylation levels upon mitochondrial stress in the presence or absence of the NuRD complex. We observed that the acetylation on histone H3, including the specific sites on H3 (K9, K14, and K27), was decreased upon *cco-1* RNAi treatment, and the decrease in histone acetylation levels was fully suppressed in *lin-40*(*yth27*) mutants, indicating that the changes in histone acetylation upon mitochondrial stress are mediated primarily through the NuRD complex (Fig. 3, C and D). In addition, supplementation of sodium butyrate (*27*), a class I histone deacetylase inhibitor, prevented the decrease in histone acetylation levels and mitochondrial stress–induced chromatin reorganization in a dose-dependent manner (Fig. 3, E and F, and fig. S3, B to E). To avoid the influence of bacterial growth on drug treatment experiments, we also confirmed our results with worms grown on bacteria arrested by kanamycin treatment (fig. S3, F and G). Together, these results indicate that the NuRD complex is required for the mitochondrial stress–induced chromatin reorganization.

### Effects of acetyl-CoA abundance on histone acetylation and chromatin structure

**Fig. 4:**
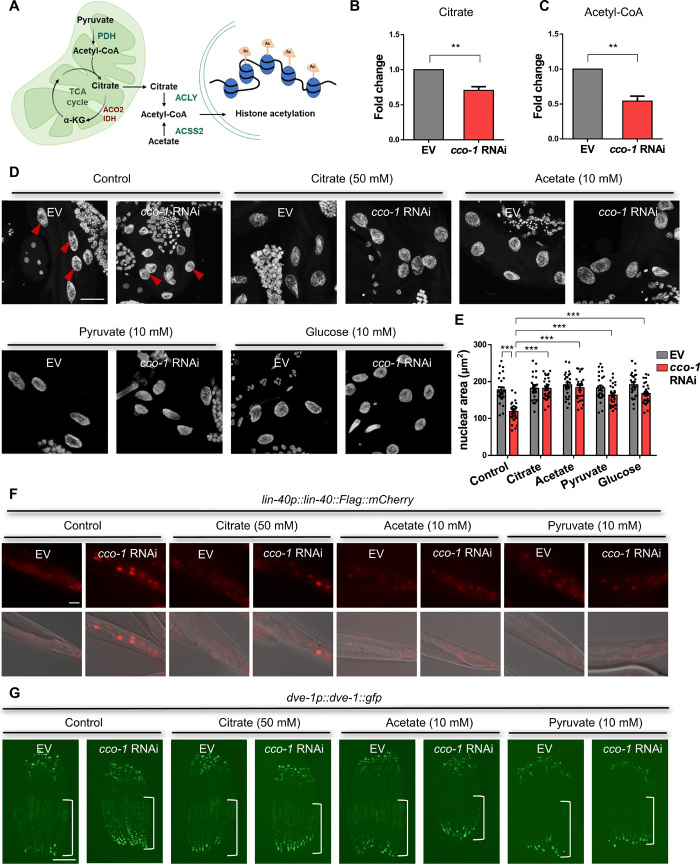
Effects of acetyl-CoA abundance on chromatin structure and the NuRD complex in response to mitochondrial stress. (**A**) Illustration diagram showing that acetyl-CoA availability is nutrient dependent and dynamically regulates histone acetylation levels. Presence of pyruvate, citrate, and acetate contributes to the production of acetyl-CoA for histone acetylation. (**B**) Citrate concentration measurement of L4 worms grown on EV or *cco-1* RNAi bacteria using whole-worm lysate. *n* = 3. (**C**) Acetyl-CoA concentration measurement of L4 worms grown on EV or *cco-1* RNAi bacteria using whole-worm lysate. *n* = 3. (**D**) Representative maximal intensity projection images of DAPI immunostaining of intestinal nuclei in day-1 adult animals grown on EV or *cco-1* RNAi bacteria with or without citrate (50 mM), acetate (10 mM), pyruvate (10 mM), or glucose (10 mM) treatment. DAPI (grey). The red arrow indicates the intestinal nucleus. Scale bar, 25 μm. (**E**) Quantification of the intestinal nuclear maximum cross-section area at day 1 of adulthood in animals as shown in (D). *n* ≥ 25 nuclei. (**F**) Representative photomicrographs of *lin-40p::lin-40::Flag::mCherry* animals grown on EV or *cco-1* RNAi bacteria with or without different compounds treatment (as indicated). Pictures were taken at day 1 of adulthood. Scale bar, 25 μm. (**G**) Representative photomicrographs of *dve-1p::dve-1::gfp* animals grown on EV or *cco-1* RNAi bacteria with or without different compounds treatment (as indicated). Pictures were taken at day 1 of adulthood. The posterior region of the intestine where DVE-1::GFP is induced or suppressed is highlighted with a white line. Scale bar, 250 μm. **P < 0.01 and ***P < 0.0001 via *t* test. Error bars, SEM.

To study how mitochondrial stress induces NuRD-dependent epigenetic modifications, we hypothesized that major metabolic changes upon mitochondrial stress could function as the signal for the NuRD-mediated chromatin reorganization. Mitochondrial impairment could result in altered levels of many metabolic intermediates; however, the availability of the metabolite acetyl-CoA was shown to be rate limiting for histone acetylation (*28*). Acetyl-CoA can be produced from various sources inside mitochondria, cytosol, and nucleus in various cell types among different organisms (*29*). Mitochondrial acetyl-CoA can be generated through several pathways, including a pyruvate derived from glycolysis that enters into mitochondria and is converted to acetyl-CoA by pyruvate dehydrogenase (PDH) complex (*pdha-1* and *pdhb-1*) (*30*). Because mitochondrial acetyl-CoA cannot pass through mitochondrial membranes, it is transferred from mitochondria to the cytoplasm in the form of citrate (a TCA cycle intermediate), where it is converted back to acetyl-CoA by ATP-citrate lyase (ACLY, *acly-1*) (*19*). In addition to ACLY-1, another major source of acetyl-CoA can be produced outside mitochondria from acetate by acyl-CoA synthetase short-chain family member 2 (ACSS2, *acs-19*) in both the cytosol and nucleus (Fig. 4A) (*31*, *32*). These reports suggested that decreased ETC activity and reduced TCA flux may decrease the level of citrate, which accounts for the reduced production of acetyl-CoA and thus causes the observed decrease in histone acetylation and changes in chromatin structure.

To test this hypothesis, we first measured the level of citrate and acetyl-CoA in animals with or without *cco-1* RNAi treatment. Both citrate and acetyl-CoA levels were significantly decreased in animals with *cco-1* RNAi treatment (Fig. 4, B and C). To further confirm that the level of acetyl-CoA is the key metabolite that mediates changes in chromatin structure, we supplemented the substrates and nutrients required for acetyl-CoA production to animals during development with or without *cco-1* RNAi treatment and examined their chromatin structure. Supplementation of citrate, acetate, pyruvate, and glucose during development compensated for the decrease of acetyl-CoA and restored histone acetylation level and strongly attenuated the chromatin reorganization under mitochondrial stress conditions on both normal and kanamycin-arrested bacteria, whereas supplementation of these nutrients during adulthood had no effect on chromatin structure (Fig. 4, D and E; and figs. S4, A to H, and S5, A to F). Supplementation of citrate, acetate, and pyruvate also strongly inhibited the nuclear accumulation of the NuRD subunit LIN-40::mCherry and the DVE-1::GFP upon *cco-1* RNAi treatment (Fig. 4, F and G), indicating that the metabolite acetyl-CoA functions upstream of the chromatin modifiers to mediate the chromatin structure upon mitochondrial stress.

### Reduction of acetyl-CoA via genetic manipulations causes the chromatin structure changes mimicking mitochondrial stress

To further demonstrate that the decreased level of acetyl-CoA results in the chromatin remodeling via NuRD complex, we first investigated how the metabolic pathways involved in the production of intracellular acetyl-CoA are regulated in response to mitochondrial stress. We observed that both PDHA-1 and ACLY-1 are significantly up-regulated upon *cco-1* RNAi, while ACS-19 is down-regulated, indicating that the production of mitochondrial-derived acetyl-CoA by ACLY-1 may predominate in the intestine of *C. elegans* (fig. S6, A to C). ACSS2 and PDH nuclear translocation have been reported to regulate local acetyl-CoA production and histone acetylation level (*30*, *31*). However, no obvious differences in the subcellular distribution were observed for either ACS-19 or PDHA-1 under *cco-1* RNAi conditions in *C. elegans* (fig. S6, A to C). Nonetheless, ACS-19 and PDHA-1 also participate in generating acetyl-CoA and can compensate for the loss of acetyl-CoA when their substrates are available. Therefore, we found that supplementation of citrate, acetate, and pyruvate counteracted the chromatin structure change upon *cco-1* RNAi treatment in an *acly-1*, *acs-19*, and *pdh* (*pdha-1* and *pdhb-1*) dependent manner, respectively (fig. S6, D to I). In addition, inhibition of aconitase dehydratase (*aco-2*) (*33*), which catalyzes the stereo-specific isomerization of citrate to isocitrate, results in accumulation of citrate and, in turn, also suppressed chromatin reorganization as well as the nuclear accumulation of DVE-1::GFP upon *cco-1* RNAi treatment (fig. S6, J to L).

**Fig. 5:**
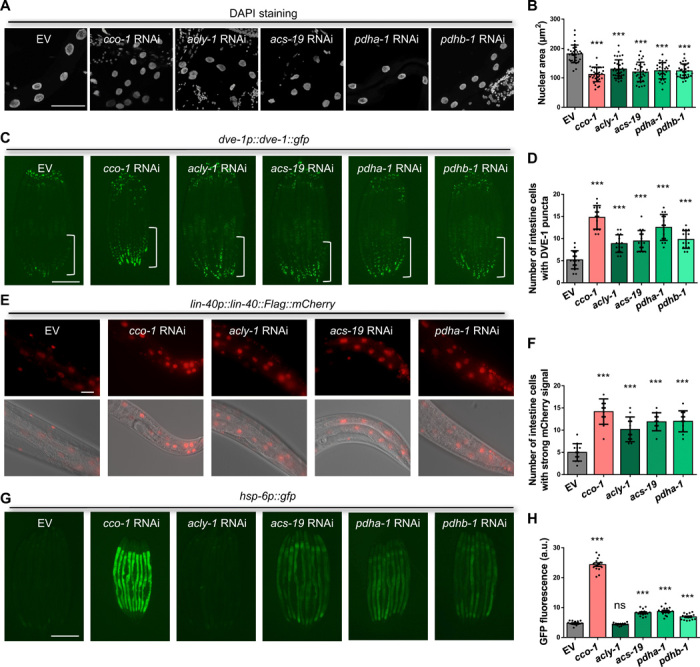
Reduction of acetyl-CoA synthesis causes chromatin structure changes mimicking mitochondrial stress. (**A**) Representative maximal intensity projection images of DAPI immunostaining of intestinal nuclei in day-1 adult WT animals grown on EV, *cco-1*, *acly-1*, *acs-19*, *pdha-1*, or *pdhb-1* RNAi bacteria from hatch. DAPI (grey). Scale bar, 50 μm. (**B**) Quantification of the intestinal nuclear maximum cross-section area at day 1 of adulthood in animals as shown in (A). *n* ≥ 30 nuclei. (**C**) Representative photomicrographs of *dve-1p::dve-1::gfp* animals grown on EV, *cco-1*, *acly-1*, *acs-19*, *pdha-1*, or *pdhb-1* RNAi bacteria from hatch. The posterior region of the intestine where DVE-1::GFP is induced or suppressed is highlighted with a white line. Scale bar, 250 μm. (**D**) Quantification of the number of intestinal nuclei with GFP signal in animals, as shown in (C). *n* = 15 worms. (**E**) Representative photomicrographs of *lin-40p::lin-40::Flag::mCherry* animals grown on EV, *cco-1*, *acly-1*, *acs-19*, or *pdha-1* RNAi bacteria from hatch. Scale bar, 25 μm. (**F**) Quantification of the number of intestinal nuclei with strong mCherry signal in animals, as shown in (E). *n* ≥ 10 worms. (**G**) Representative photomicrographs of *hsp-6p::gfp* animals grown on EV, *cco-1*, *acly-1*, *acs-19*, *pdha-1*, or *pdhb-1* RNAi bacteria from hatch. Scale bar, 250 μm. (**H**) Quantification of *hsp-6p::gfp* expression level of the entire intestine in animals, as shown in (G). *n* = 15 worms. ***P < 0.0001 and ns denotes *P* > 0.05 via *t* test. Error bars, SEM. a.u., arbitrary units.

Moreover, we found that animals lacking cytosolic ACLY-1 enzyme, which restricts the production of cytosolic acetyl-CoA from citrate, are sufficient to induce global chromatin reorganization, nuclear accumulation of DVE-1, and the NuRD subunit LIN-40 even under normal conditions without mitochondrial stress (Fig. 5, A to F). Similarly, RNAi against the enzymes *pdha-1* and *acs-19*, which are involved in acetyl-CoA production, also mimics the phenotype of decreased acetyl-CoA upon mitochondrial impairment, with a minor effect on mitochondrial function by monitoring the expression levels of the UPR^mt^ reporter *hsp-6p::gfp* (Fig. 5, A to H). Collectively, these results indicate that acetyl-CoA is a central metabolic intermediate that coordinates the mitochondrial metabolic status and the epigenome via the NuRD complex in *C. elegans*.

### Dietary nutrients diminish mitochondrial stress–induced longevity

**Fig. 6:**
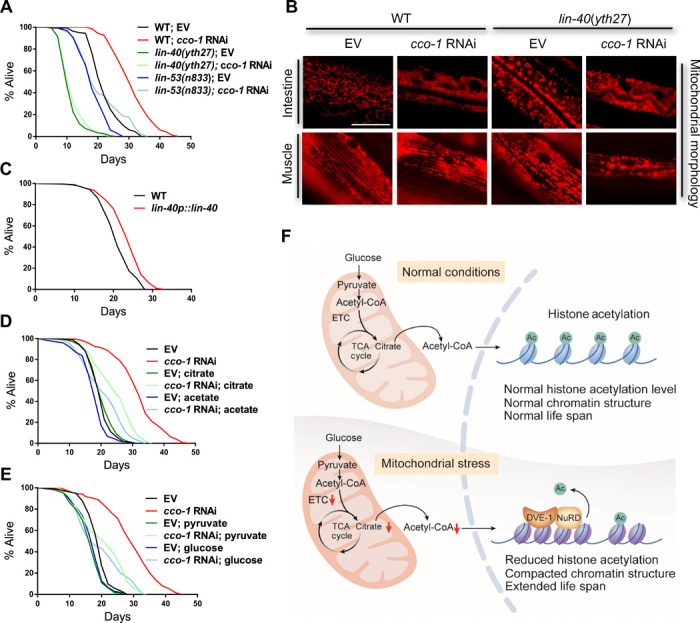
Dietary nutrients diminish mitochondrial stress–induced longevity. (**A**) Survival analyses of WT, *lin-40*(*yth27*), and *lin-53*(*n833*) animals on EV or *cco-1* RNAi bacteria. (**B**) Representative photomicrographs of *ges-1p::tomm-20::HA::mKate2* and *myo-3p::tomm-20::HA::mKate2* animals in a WT or *lin-40* mutant background grown on EV or *cco-1* RNAi bacteria from hatch. Pictures were taken at day 1 of adulthood. Scale bar, 25 μm. (**C**) Survival analyses of WT and LIN-40 overexpression animals on OP50 bacteria. (**D**) Survival analyses of WT animals on EV or *cco-1* RNAi bacteria with or without citrate (50 mM) or acetate (10 mM) treatment. (**E**) Survival analyses of WT animals on EV or *cco-1* RNAi bacteria with or without pyruvate (10 mM) or glucose (10 mM) treatment. (**F**) Model of acetyl-CoA links mitochondrial stress to longevity via NuRD-mediated chromatin remodeling. Under nonstressed conditions, acetyl-CoA can be generated from citrate and acts as the substrate for histone acetylation. Under these conditions, DVE-1 and the NuRD complex do not accumulate in the nucleus, animals have normal life span, and nucleus is not compacted. During mitochondrial stress, decreased citrate level results in limited production of acetyl-CoA, leading to the accumulation of DVE-1 and NuRD in the nucleus. Consequently, the histone acetylation level is decreased, nuclei become compacted, and animals are long-lived.

To assess the potential role of the NuRD complex in mediating the mitochondrial stress–induced longevity in *C. elegans*, we examined the life span of animals lacking NuRD subunits with or without mitochondrial stress. Although both *lin-40*(*yth27*) and *lin-53*(*n833*) animals were short-lived under normal conditions, these mutants completely suppressed the *cco-1* RNAi–induced life-span extension (Fig. 6A; see table S1 for life-span statistics). *lin-40* mutants showed perturbed mitochondrial morphology, and their morphology worsened in the combination of *cco-1* RNAi treatment in both muscle and intestinal cells (Fig. 6B), further demonstrating the role of the NuRD complex in maintaining mitochondrial homeostasis. Furthermore, overexpression of the MTA/LIN-40, the scaffold protein for NuRD complex assembly (*34*), modestly but consistently increased life span in *C. elegans* (Fig. 6C; see table S1 for life-span statistics).

To understand the potential role of *lin-40* in regulating the life-span extension during mitochondrial stress, we performed RNA sequencing (RNA-seq) analyses of WT and *lin-40*(*yth27*) animals, each in the presence or absence of *cco-1* RNAi. Our analyses showed that majority of the genes differentially expressed in WT animals upon *cco-1* RNAi treatment were abrogated in *lin-40*(*yth27*) mutant animals, especially the down-regulated genes involved in the metabolic processes (fig. S7, A and B). Genes in the OXPHOS, TCA cycle, and glycolysis pathway that were either down-regulated or not significantly changed in *cco-1* RNAi–treated WT animals were significantly up-regulated in *lin-40* mutant animals upon *cco-1* RNAi treatment (fig. S7C). Likewise, the down-regulation of the ribosome subunits in response to *cco-1* RNAi was also abolished in *lin-40* mutant animals (fig. S7 D to G). These results suggest that the role of the NuRD complex in the regulation of mitochondrial stress response is consistent with its conserved function as a transcriptional repressor (*35*, *36*).

To assess whether excessive supplementation of nutrients to restore acetyl-CoA levels affects the mitochondrial stress–induced life-span extension, we examined the life span of *cco-1* RNAi–treated animals with supplementation of citrate, acetate, pyruvate, and glucose. The extended life span of worms upon mitochondrial ETC inhibition was diminished when acetyl-CoA levels were restored by adding substrates and nutrients (Fig. 6, D and E; see table S1 for life-span statistics). However, the reduced body size and the decreased brood size or oxygen consumption due to mitochondrial dysfunction were not restored by the same treatment (fig. S8, A to C). Together, our results indicate that animals could coordinate the cellular metabolic changes and input of the dietary nutrients during development to shape the chromatin structure, leaving a durable epigenetic signature that ultimately determined the aging process (Fig. 6F).

## DISCUSSION

In this study, we found that mitochondrial perturbations alter the nuclear epigenome that determines the organismal life span, a process mediated by the NuRD complex in responding to the availability of the metabolite acetyl-CoA. The abundance of the cellular acetyl-CoA level represents the general energetic state of the cells during growth and development; fluctuations in acetyl-CoA level have the capacity to modulate histone acetylation, resulting in epigenetic adaptation to environmental stress conditions (*29*). Here, we identified that the NuRD complex responds to the cellular level of acetyl-CoA to reorganize chromatin structure for a specific transcriptional program essential for life-span extension.

Alterations in chromatin structure have been observed as signs during both physiological aging and premature aging syndromes (*37*). Loss of the NuRD complex seems to be an early event during aging, which contributes to alterations in the chromatin structure, making the genome more susceptible to DNA damage (*38*). Our study further demonstrated that the loss of the NuRD subunit LIN-40 is an early event during aging, and promoting NuRD complex accumulation in the nucleus is important to maintain chromatin structure and induce life-span extension caused by mitochondrial perturbations. The mechanism by which acetyl-CoA mediates NuRD nuclear accumulation is yet to be determined.

Under certain circumstances, stress experienced early in life can induce a long-lasting adaptive stress response that may even influence the progression of the aging process (*39*). In *C. elegans*, mild mitochondrial stress experienced during early development induces life-span extension through an epigenetic mechanism mediated by an epigenetic factor LIN-65 and histone H3K9 methyltransferase MET-2 to ensure that the beneficial effects are maintained into later life (*11*). In this study, we found that the metabolite acetyl-CoA level represents a critical mitochondrial stress signal that mediate the NuRD complex to restructure the chromatin, leaving durable epigenetic modifications that may counteract the aging process. It is therefore tempting to speculate that chromatin is more receptive to acetyl-CoA–induced epigenetic modifications in early life and that the NuRD complex may coordinate with LIN-65/MET-2–mediated histone modifications to establish an epigenetic memory that persists into adulthood.

As mitochondria are central in the regulation of cellular energy and metabolic homeostasis, mitochondrial dysfunctions significantly contribute to the aging process. Emerging evidences showed that activation of hypoxia-inducing TF HIF-1, increased the level of reactive oxygen species (ROS), and induction of the UPR^mt^ could promote mitochondrial homeostasis and mediate life-span extension (*4*, *40*). It remains unclear whether multiple distinct mechanisms are interconnected to mediate enhanced longevity following various mitochondrial stresses (*41*). Our studies uncovered that reduced production of acetyl-CoA due to mitochondrial perturbations elicits global histone deacetylation via the NuRD complex to mediate stress-induced longevity. Although the extended life span of worms upon mitochondrial ETC inhibition was diminished when acetyl-CoA levels were restored by adding substrates and nutrients, the reduced body/brood size or oxygen consumption rate was not restored by the same treatments, indicating that the functions of mitochondria in ATP production, metabolic regulation, and ROS signaling on development and the aging process are distinct. Mitochondrial perturbations may act via highly specific mechanisms to induce a distinct stress response pathway best suited to the cells and organisms (*42*). It maybe thus possible to obtain the benefits to life-span extension of reduced mitochondrial respiration without incurring a reduction in fecundity and body size, through a suitable intervention of nutrients in the diet during early life.

## MATERIALS AND METHODS

### *C. elegans* maintenance and transgenic lines

Nematodes *C. elegans* were maintained and experimentally examined at 20°C on a standard nematode growth medium (NGM) agar plates seeded with *Escherichia coli* OP50 or HT115. *E. coli* OP50 was cultured overnight in LB at 37°C. *E. coli* HT115 was used to perform RNAi and was cultured overnight at 37°C in LB containing carbenicillin (100 μg/ml). Strains used in this study were listed in table S2.

### Transgenic strain construction

The *dve-1* 1.8-kb promoter was used to generate *dve-1p::mCherry*; the *lin-40* 2.0-kb promoter and *lin-40* complementary DNA (2610 bp) were used to generate *lin-40p::lin-40::Flag::mCherry*; the *acly-1* 2.5-kb promoter and *acly-1* 4513-bp sequence were used to generate *acly-1p::acly-1::mCherry*; the *acs-19* 2.0-kb promoter and *acs-19* 2323-bp sequence were used to generate *acs-19p::acs-19::mCherry*; and the *pdha-1* 2.0-kb promoter and *pdha-1* 1321-bp sequence were used to generate *pdha-1p::pdha-1:mCherry*. mCherry was from pBR322 backbone and the sequence of Flag was synthesized. An ABclonal MultiF Seamless Assembly Mix was used for cloning.

Transgenic strains were generated by microinjecting target constructs (50 ng/μl) mixed with a pRF4(*rol-6*) (50 ng/μl) coinjection marker. Integrated lines were generated using ultraviolet irradiation and outcrossed six times.

### DVE-1 antibody

The DVE-1 polyclonal antibody was raised in rabbit by immunization with a fragment of DVE-1 (153 to 392 amino acids), which was expressed as a His6-SUMO–tagged fusion protein in *E. coli*. The serum was subjected to antigen-specific antibody purification. Purified antibody was used in Western blots at a dilution of 1:5000. Worms should be washed several times with M9 before immunoblotting to reduce background bands caused by bacterial contamination.

### RNAi feeding

RNAi bacteria were grown in LB media containing carbenicillin (100 μg/ml) at 37°C overnight. The next day, RNAi bacteria were induced with 1 mM isopropyl-β-D-thiogalactopyranoside (IPTG) for another 4 hours at 37°C and then seeded on NGM plates supplemented with carbenicillin (100 μg/ml) and 1 mM IPTG. Age-synchronized worms were bleached and grown from hatch on *E. coli* HT115 strains containing an empty vector (EV) control or double-stranded RNA. RNAi strains were from the Vidal library, if present, or the Ahringer library, if they were absent from the Vidal library.

### Microscopy and image analysis

#### Analysis of the fluorescence intensity in whole worm

For whole-animal fluorescence image, worms were anesthetized with 50 mM sodium azide and imaged using a Leica M165 FC dissecting microscope. To quantify fluorescent intensity, the entire intestine regions were outlined and quantified using ImageJ software. For quantification of the DVE-1::GFP, LIN-40::mCherry, and LIN-53::GFP nuclear localization, the worms were mounted on 2% agarose pads with 50 mM sodium azide, and photographs were taken using Zeiss Imager M2 microscope.

#### Dissection and fixation for intestinal nuclei analysis

Dissection and fixation were carried out by a modified freezing-crack method (*43*). Worms were dissected in M9 buffer on a polylysine-coated slide. The slides were fixed in methanol for 10 min, followed by acetone for 10 min at −20°C. Next, the slides were placed in phosphate-buffered saline (PBS) and washed with fresh PBS for 5 min. Slides were then mounted with 4′,6-diamidino-2-phenylindol (DAPI) solution (VECTASHIELD). We used at least 30 worms in each sample for DAPI staining and intestinal nuclei from more than 10 worms were selected and quantified for nuclear size.

#### Maximum cross-section area analysis of intestinal nuclei

Intestinal cells were imaged at ×63 magnification using Leica-TCS SP8, laser scanning confocal microscope. Z-stacks of the intestine were analyzed using LAS X. Contours of individual nuclei, according to DAPI fluorescence, were manually defined and used to render nuclei area. Maximum cross-section area of each nucleus was acquired from the surface tool statistics by Image J.

### Biochemistry

#### IP-MS and coimmunoprecipitation

Synchronized day-1 adult worms grown on plates were collected and washed in M9 buffer. After washing, the animals were resuspended in 0.5 volumes of extraction buffer [50 mM tris-HCl, (pH 7.4), 150 mM NaCl, 5 mM dithiothreitol, 10% glycerol, 0.1% NP-40, and protease inhibitors]. The suspension was then dripped into liquid N_2_, and the resulting balls were grinded using mortar and pestle. The homogenized worm tissue was resuspended with 2 volumes of extraction buffer and lysed at 4°C for 30 min, and the insoluble materials were then removed by centrifugation at 13,000 rpm at 4°C.

For IP-MS, 10 ml of lysate was mixed with GFP-trap agarose (Chromotek) (100 μl) for 4 hours and washed for five times with wash buffer [50 mM tris-HCl, (pH 7.4), 150 mM NaCl, 10% glycerol, and protease inhibitors]. Proteins were eluted using 1% SDS by boiling and then subjected to MS analysis. See data file S1 for the list of genes identified in the IP-MS experiments (related to Fig. 1A).

For coimmunoprecipitation, 2 ml of lysate was mixed with FLAG M2 Affinity Gel (Sigma-Aldrich) (20 μl) for 4 hours. Immunoprecipitate was washed for four times with wash buffer [50 mM tris-HCl, (pH 7.4), 150 mM NaCl, 10% glycerol, and protease inhibitors]. Samples were then subjected to Western blot analysis.

#### Western blot analysis

For large-scale experiments, age-synchronized worms were washed off from the plate with M9 buffer and frozen in liquid nitrogen. Worm extracts were generated by sonication using Covaris ME220 in nondenaturing lysis buffer [150 mM NaCl, 50 mM tris-HCl, (pH 8.0), 1 mM EDTA, 1% Triton X-100, and protease inhibitor]. For small-scale experiments, 100 to 150 worms were picked into 16 μl of M9 buffer and frozen in liquid nitrogen. Before running the Western blot, 5× SDS loading buffer was added to each sample, mixed well, and boiled for 15 min and resolved by SDS–polyacrylamide gel electrophoresis (Bio-Rad). Antibodies used in this study were listed in data file S3.

#### Acetyl-CoA measurement

The extraction of acetyl-CoA from worms was carried out, as previously described with some modifications (*44*). Briefly, synchronized L4 stage worms were washed off from the plates with M9 buffer, and the worm pellet was repeatedly washed with dH_2_O for three times followed by overnight freeze-drying in a 2-ml Sarstedt tube. The sample was weighed then resuspended with 300 μl of extraction buffer containing isopropanol, 50 mM KH_2_PO_4_, and bovine serum albumin (50 mg/ml) [25:25:1 (v/v/v)], acidified with the addition of glacial acetic acid. Next, 19:0 CoA was added as an internal standard, and the tissues were homogenized on a bead ruptor (Omni) under the conditions optimized for muscles (8 m/s, 8 s, two cycles, pause = 5 s). Following homogenization, 300 μl of petroleum ether was added, and the sample was centrifuged at 9000 rpm for 2 min at 4°C. The upper phase was removed. The samples were extracted two more times with petroleum ether as described above. To the lower phase lastly remaining, 5 μl of saturated ammonium sulfate was added followed by 600 μl of chloroform:methanol [1:2 (v/v)]. The sample was then incubated on a thermomixer at 450 rpm for 20 min at 25°C, followed by a centrifuge at 12,000 rpm for 5 min at 4°C. Clean supernatant was transferred to fresh tube and subsequently dried in the SpeedVac under OH mode (Genevac). Dry extracts were resuspended in appropriate volume of methanol:water [9:1 (v/v)] before liquid chromatography–MS analyses (Agilent).

#### Citrate measurement

Synchronized L4 stage worms were collected and washed with M9 buffer and Milli-Q water. Citrate content was measured using a citrate assay kit (Sigma-Aldrich, MAK057). Briefly, about 30 μl of worms was homogenized with 100 μl of citrate assay buffer using Covaris ME220. About 10 μl of homogenate was subjected to protein concentration measurement by Bradford assay (Beyotime, P0006C), and the rest was used in the citrate assay according to the manufacturer’s protocol.

### Compound treatment assay

#### Sodium butyrate treatment

Synchronized L1 stage worms were transferred to an S-medium containing sodium butyrate. Worms were placed on an orbital shaker at 200 rpm at 20°C and monitored for 48 hours before analysis.

#### Metabolites treatment

We performed metabolites treatment assays by adding citrate, acetate, pyruvate, or d-glucose to the liquid culture medium. Worms were grown on plates after hatch and transferred to a liquid culture medium with HT115 or kanamycin-arrested HT115 RNAi bacteria containing metabolites from L1 or day-1 adulthood. The cultures were incubated on orbital shaker at 200 rpm at 20°C and continuously monitored for 48 hours before analysis.

### Life-span analysis

Life-span experiments were performed on NGM plates at 20°C, as previously described (*7*). To prevent progeny production, 100 μl of 5-fluoro-2′-deoxyuridine (FUdR; 10 mg/ml) was added to seeded plates. Worms were synchronized by egg bleach and were grown on HT115 RNAi bacteria from hatch and transferred to FUdR plates from L4. For life-span experiments with the addition of metabolites, synchronized L1 worms grown on HT115 RNAi plates for 1 day were transferred to a liquid culture medium with HT115 RNAi bacteria containing corresponding metabolites or equal volume of deionized water (all the metabolites we used in this study are resolved in water). The cultures were incubated on orbital shaker at 200 rpm at 20°C for 48 hours until worms reached to adult stage. Then, worms were washed for three times and placed on FUdR plates seeded with OP50 not HT115 RNAi bacteria (*cco-1* RNAi during development only is sufficient to induce life-span extension). Worms were scored every second day from day-1 adult stage. All life-span data are available in extended data of table S1. Prism6 software was used for statistical analysis. Log-rank (Mantel-Cox) method was used to determine the significance difference.

### Statistical analysis

All experiments were repeated at least two times with identical or similar results. Data represent biological replicates. Appropriate statistical tests were used for every figure. Data met the assumptions of the statistical tests described for each figure. Statistical parameters, including the exact value of *n* and descriptive statistics (mean ± SEM) and statistical significance, are reported in the figures and the figure legends. Data are judged to be statistically significant when *P* < 0.05 by two-tailed Student’s *t* test. In figures, asterisks denote statistical significance as calculated by Student’s *t* test (*P < 0.05, **P < 0.01, and ***P < 0.0001), as compared to appropriate controls.

## Supplementary Material

abb2529_Data_file_S2.xlsx

abb2529_Data_file_S3.xlsx

abb2529_SM.pdf

abb2529_Data_file_S1.xlsx

## SUPPLEMENTARY MATERIALS

Supplementary material for this article is available at http://advances.sciencemag.org/cgi/content/full/6/31/eabb2529/DC1

## Appendix

View/request a protocol for this paper from *Bio-protocol*.
