# Supplementary material for: NuRD mediates mitochondrial stress–induced longevity via chromatin remodeling in response to acetyl-CoA level
Source: Sci Adv. 2020 Jul 31;6(31):eabb2529. doi: 10.1126/sciadv.abb2529 (PMC7400466; doi:10.1126/sciadv.abb2529)
Supplement: abb2529_SM.pdf [file abb2529_SM.pdf]

Science Advances
AAAS

advances.sciencemag.org/cgi/content/full/6/31/eabb2529/DC1

Supplementary Materials for

NuRD mediates mitochondrial stress–induced longevity via chromatin remodeling in response to acetyl-CoA level

Di Zhu, Xueying Wu, Jun Zhou, Xinyu Li, Xiahe Huang, Jiasheng Li, Junbo Wu, Qian Bian, Yingchun Wang, Ye Tian*

*Corresponding author. Email: ytian@genetics.ac.cn (Y.T.)

Published 31 July 2020, *Sci. Adv.* **6**, eabb2529 (2020)
DOI: 10.1126/sciadv.abb2529

The PDF file includes:

Supplementary Materials and Methods
Fig. S1 to S8
Tables S1 and S2

Other Supplementary Material for this manuscript includes the following:

(available at advances.sciencemag.org/cgi/content/full/6/31/eabb2529/DC1)

Data files S1 to S3

Materials and Methods

EMS mutagenesis screen

~100 L4 worms were washed with M9 buffer for three times and suspended in 3 ml M9 buffer. 20 µl EMS (Ethyl Methane sulfonate, Sigma #M-0880) was added into 1ml M9 buffer. The 3 ml worms were transferred into the 1ml EMS solution making the final concentration of EMS to 47 mM and incubated at 20°C on a spinning wheel for 4 hours. Then, worms were washed with M9 and transferred to plates. Healthy looking late L4 animals were picked off to use as P0. F1 progeny were then allowed to self-fertilized, and F2 animals were screened for the phenotype of interest.

Heat shock assay

Synchronized day 1 adult worms of different genetic backgrounds were incubated in 34°C for 20 minutes.

ER stress assay

Synchronized L4 stage worms of different genetic backgrounds were incubated in M9 buffer containing 25 ng/µl tunicamycin or DMSO for 4 hours.

RNA-seq

N2 and *lin-40*(*yth27*) animals were grown from hatch at 20ºC on EV or EV+*cco-1* RNAi bacteria and collected by washing with M9 at the L4 stage, followed by snap frozen in liquid nitrogen. Total RNA was extracted from the worms using TRIzol® Reagent according the manufacturer's instructions (Invitrogen #15596018) and genomic DNA was removed using RQ1 RNase-Free DNase (Promega #M6101). Then RNA quality was determined by 2100 Bioanalyser (Agilent) and quantified using the ND-2000 (NanoDrop Technologies). Only high-quality RNA sample (OD260/280=1.8~2.2,

OD260/230≥2.0, RIN ≥ 6.5, 28S:18S ≥ 1.0, > 2μg) was used to construct sequencing library.

RNA-seq transcriptome library was prepared following TruSeqTM RNA sample preparation Kit from Illumina (San Diego, CA) using 1μg of total RNA. Shortly, messenger RNA was isolated according to polyA selection method by oligo(dT) beads and then fragmented by fragmentation buffer firstly. Secondly double-stranded cDNA was synthesized using a SuperScript double-stranded cDNA synthesis kit (Invitrogen, CA) with random hexamer primers (Illumina). Then the synthesized cDNA was subjected to end-repair, phosphorylation and 'A' base addition according to Illumina's library construction protocol. Libraries were size selected for cDNA target fragments of 200–300 bp on 2% Low Range Ultra Agarose followed by PCR amplified using Phusion DNA polymerase (NEB) for 15 PCR cycles. After quantified by TBS380, paired-end RNA-seq sequencing library was sequenced with the Illumina HiSeq-xten (2 × 150bp read length).

The raw paired end reads were trimmed and quality controlled by SeqPrep (https://github.com/jstjohn/SeqPrep) and Sickle (https://github.com/najoshi/sickle) with default parameters. Then clean reads were separately aligned to reference genome with orientation mode using TopHat (http://tophat.cbcb.umd.edu/, version 2.0.0) software. The mapping criteria of bowtie was as follows: sequencing reads should be uniquely matched to the genome allowing up to 2 mismatches, without insertions or deletions. Then the region of genes was expanded following depths of sites and the operon was obtained. In addition, the whole genome was split into multiple 15 kbp windows that share 5 kbp. New transcribed regions were defined as more than 2 consecutive windows without overlapped region of gene, where at least 2 reads mapped per window in the same orientation. To identify DEGs (differential expression genes) between two different samples, the expression level of

each transcript was calculated according to the fragments per kilobase of exon per million mapped reads (FRKM) method. RSEM (http://deweylab.biostat.wisc.edu/rsem/) was used to quantify gene abundances.

Gene ontology (GO) analysis and heat map analysis

GO analysis of the significant gene list was performed using PANTHER (http://www.pantherdb.org/), by using text files containing the Gene ID list (Data file S2). Heat maps of 33 TCA genes (cel00020), 111 OXPHOS genes (cel00190), and 42 glycolysis genes (cel00010) were generated from KEGG pathway database (http://www.genome.jp/kegg/). Fold change (FC) was calculated by comparing normalized count values of each condition to each empty vector (EV) control and then transformed to log2 scale, each condition was repeated three times. Heat maps were generated using a web tool (https://www.omicsolution.org/wkomics).

See Data file S2 for complete list of genes used to assemble heat maps.

RNA isolation and quantitative PCR analyses

Total RNA was isolated using TRIzol (Invitrogen #15596018). Worms were synchronized and washed off from the plates using M9 buffer, and 500 μl TRIzol were added to the samples and homogenized by repeated freezing and thawing using liquid nitrogen. RNA was isolated according to manufacturer's instructions. DNA was wiped off using RQ1 RNase-Free DNase (Promega #M6101). cDNA was synthesized using the M-MLV Reverse Transcriptase (Invitrogen #28025013). Gene expression levels were determined by real-time PCR using iTaq Universal SYBR Green Supermix (Bio-Rad #1725121) and Bio-Rad CFX96 Real-Time PCR Detection Systems. Relative gene expression was normalized to *act-3* (T04C12.4) mRNA levels. In each experiment, at least three biological samples were analyzed.

The primer sequences used in the quantitative PCR are listed in Data file S3.

Brood size assay

Worms were cultured in the S-medium containing citrate (50 mM), pyruvate (10 mM), D-glucose (10 mM), or equal volume of H2O (vehicle control) with HT115 *E. coli* until young adult stage and then transferred to NGM plate seeded with OP50. The worms were transferred to fresh dishes daily until they stopped laying eggs, and the number of eggs was recorded every day. Six replicates per treatment were used. The nematode brood size was determined based on the sum of total eggs laid by individual hermaphrodites.

Measurement of oxygen consumption rates

Oxygen consumption rate (OCR) measurements were performed using Seahorse XFe-96 analyser (Seahorse Bioscience). Wild-type N2 worms were cultured in the S medium containing citrate, D-glucose, or dH_2O (vehicle control) with HT115 *E. coli*. OCR was assessed on day 1 of adulthood. Worms were collected and washed for three times with M9 to get rid the traces of bacteria. Ten worms were transferred to each well of Seahorse XF-96 cell culture microplates (Seahorse Bioscience, V3-PS), containing 200 µl M9. OCR was measured fifteen cycles (7 mins/cycle) under basal conditions and normalized to the number of worms counted per well (n=100). Each measurement was repeated at least three times.

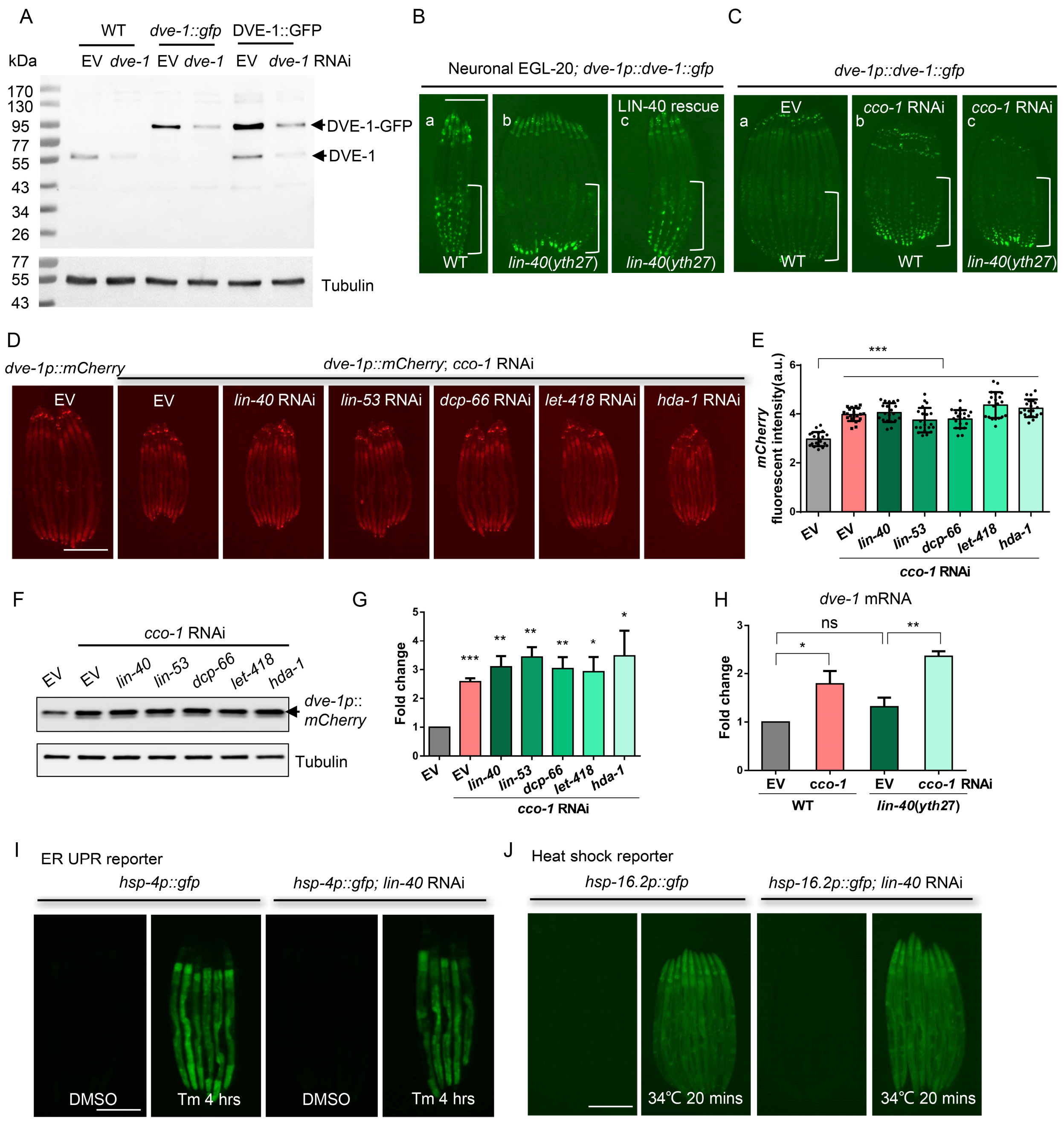

fig. S1. The NuRD complex is not required for the induction of UPR^{ER} or UPR^{cyto}. (**A**) Immunoblots of DVE-1 in WT, *dve-1::gfp* (*gfp* knock-in by CRISPR), and DVE-1::GFP (over-expression) animals grown on EV or *dve-1* RNAi bacteria using DVE-1 antibody. (**B**) Representative photomicrographs demonstrating: (a) DVE-1::GFP is accumulated in the intestinal nuclei in neuronal EGL-20 (*rgef-1p::egl-20*) animals. (b) The DVE-1::GFP nuclear accumulation was suppressed by *lin-40* mutation; (c) The suppression of DVE-1::GFP nuclear accumulation can be rescued by *lin-40p::lin-40::Flag::mCherry* expression. Scale bar, 250 μm. (**C**) Representative photomicrographs

demonstrating: (a) DVE-1::GFP expression in WT animals. (b) DVE-1::GFP is accumulated in the intestinal nuclei upon *cco-1* RNAi treatment. (c) The nuclear accumulation of DVE-1::GFP upon *cco-1* RNAi is suppressed by *lin-40* mutation. (**D**) Representative photomicrographs of *dve-1p::mCherry* animals grown on EV, *cco-1*+EV, *cco-1+lin-40*, *cco-1+lin-53*, *cco-1+dcp-66*, *cco-1+let-418*, or *cco-1+hda-1* double RNAi bacteria from hatch. Scale bar, 250 µm. (**E**) Quantification of *dve-1p::mCherry* expression of the entire intestine in animals as shown in (D). n ≥ 15 worms. (**F**) Immunoblots of mCherry in *dve-1p::mCherry* animals as shown in (D). (**G**) Quantification of mCherry protein levels of (F). n = 3. (**H**) Quantitative PCR of *dve-1* mRNA level. Synchronized L4 WT or *lin-40* mutant animals grown on EV or EV+*cco-1* RNAi bacteria from hatch were collected for qPCR. n = 3. (**I**) *lin-40* is not required for the induction of UPR^{ER} reporter *hsp-4p::gfp* expression. Synchronized L4 animals were treated with 25 ng/ml tunicamycin (Tm) or DMSO for 4 hours at 20°C prior to imaging. Scale bar, 250 µm. (**J**) *lin-40* is not required for the induction of cytosolic heat shock stress response reporter *hsp-16.2p::gfp* expression. Synchronized day 1 of adulthood animals were placed at 34°C for 20 min and allowed to recover for 6 hours prior to imaging. Scale bar, 250 µm.

*p < 0.05, **p < 0.01, ***p < 0.0001, ns denotes p > 0.05 via t-test. Error bars, SEM.

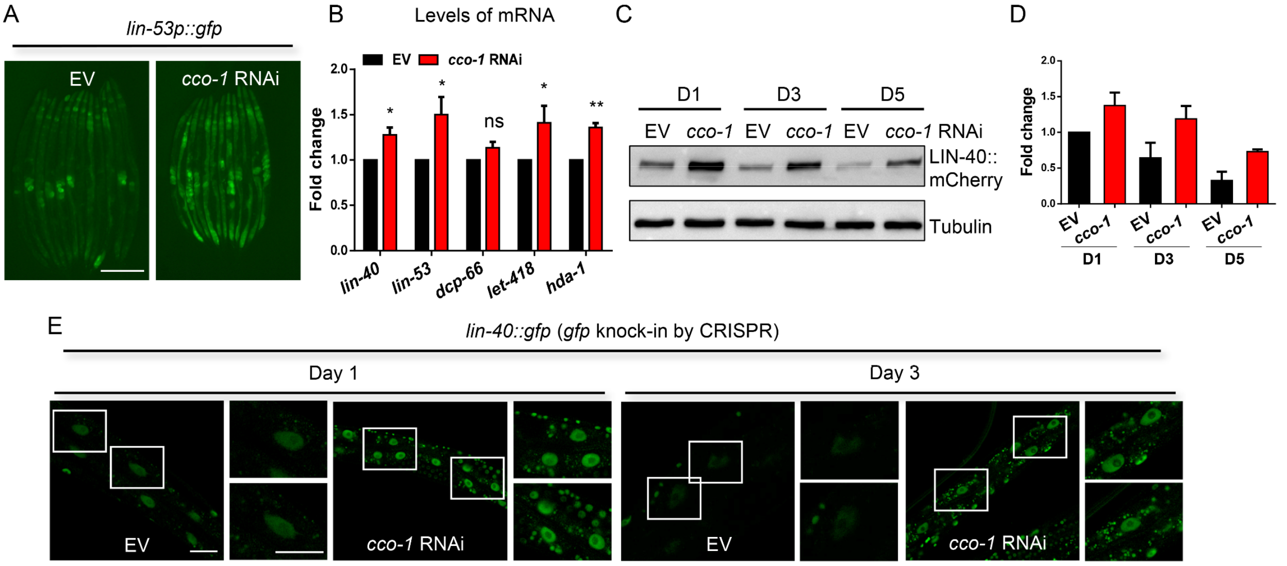

fig. S2. The expression of NuRD complex subunits is upregulated upon *cco-1* RNAi treatment. (**A**) Representative photomicrographs of *lin-53p::gfp* animals in a WT background grown on EV or *cco-1* RNAi bacteria from hatch. Scale bar, 250 µm. (**B**) Quantitative PCR of *lin-40*, *lin-53*, *dcp-66*, *let-418,* and *hda-1*mRNA level. Synchronized L4 worms grown EV or *cco-1*+EV RNAi from hatch were collected for qPCR. *p < 0.05, **p < 0.01, ns denotes p > 0.05 via t-test. Error bars indicates the SEM from three biological replicates. (**C**) Immunoblots of LIN-40::mCherry in WT animals grown on EV or *cco-1* RNAi from hatch. Animals were collected at days 1, 3, and 5 of adulthood. Anti-tubulin serves as a loading control. (**D**) Quantification of LIN-40::mCherry protein levels of (C). n = 3. (**E**) Representative photomicrographs of *lin-40::gfp* animals grown on EV or *cco-1* RNAi bacteria from hatch. Animals were observed at days 1 and 3 of adulthood. Dash rectangles highlight the areas enlarged and shown on the right. Scale bar, 25 µm.

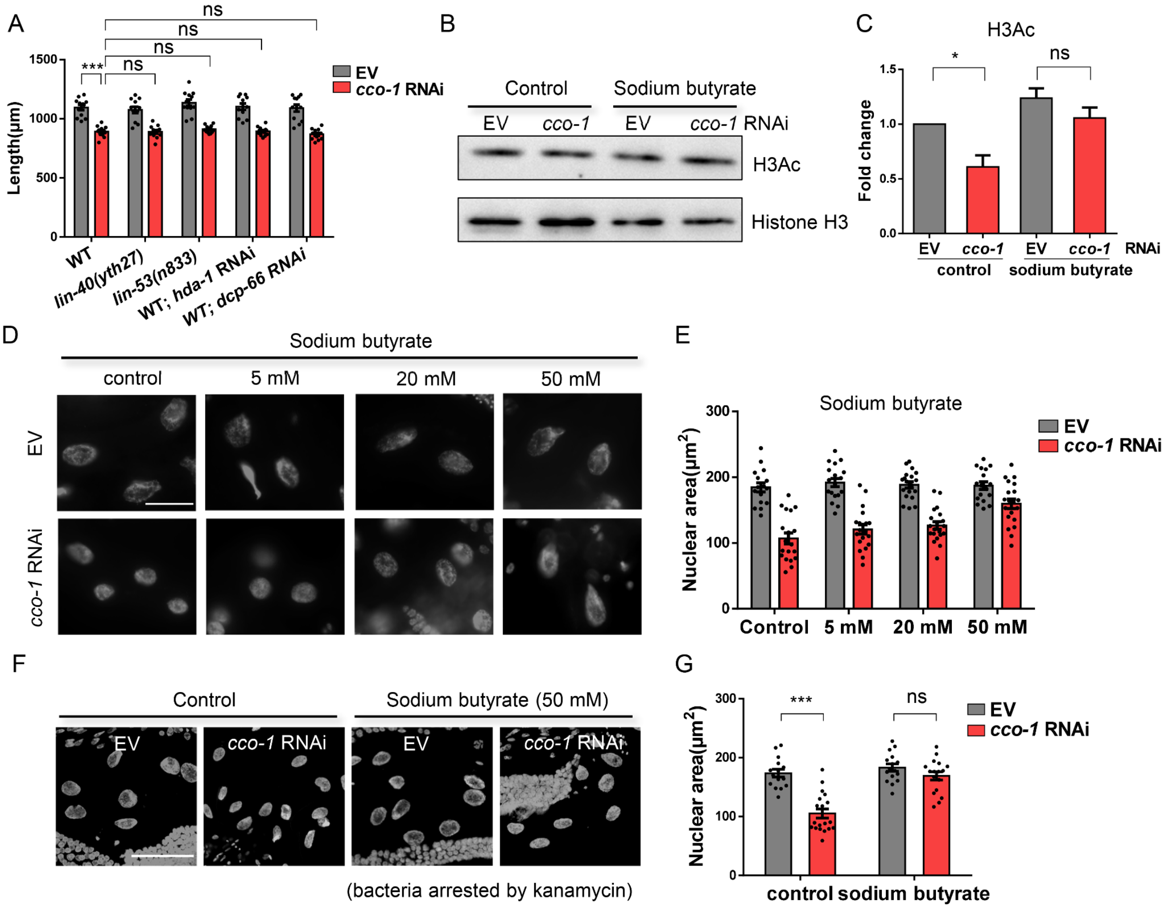

fig. S3. Histone deacetylation inhibitor suppresses nuclear reorganization under mitochondrial stress conditions. (**A**) The body length of day 1 adult WT, *lin-40*, or *lin-53* mutant animals grown on EV, EV+*cco-1*, EV+*hda-1*, *cco-1*+*hda-1*, EV+*dcp-66*, or *cco-1*+*dcp-66* double RNAi bacteria from hatch. n ⩾ 10 worms. (**B**) Immunoblots of histone H3Ac in WT animals grown on EV or *cco-1* RNAi bacteria with or without sodium butyrate treatment. (**C**) Quantification of histone H3Ac levels (relative to histone H3). n = 3. (**D**) Representative images of DAPI immunostaining of intestinal nuclei in day 1 adult animals grown on EV or *cco-1* RNAi with or without sodium butyrate treatment (5 mM, 20 mM, and 50 mM). DAPI (grey); Scale bar, 25 μm. (**E**) Quantification of the intestinal nuclear maximum cross section area at day 1 of adulthood in animals as shown in (D). n ⩾ 15 nuclei. (**F**) Representative maximal intensity projection images of DAPI immunostaining of intestinal nuclei in day 1 adult animals grown on EV or *cco-1* RNAi bacteria arrested by kanamycin with or without sodium butyrate treatment. DAPI (grey); Scale bar, 50 μm. (**G**) Quantification of the intestinal nuclear maximum cross section area at day 1 of adulthood in animals as shown in (F). n ≥ 15 nuclei.

*p < 0.05, ***p < 0.0001, ns denotes p > 0.05 via t-test. Error bars, SEM.

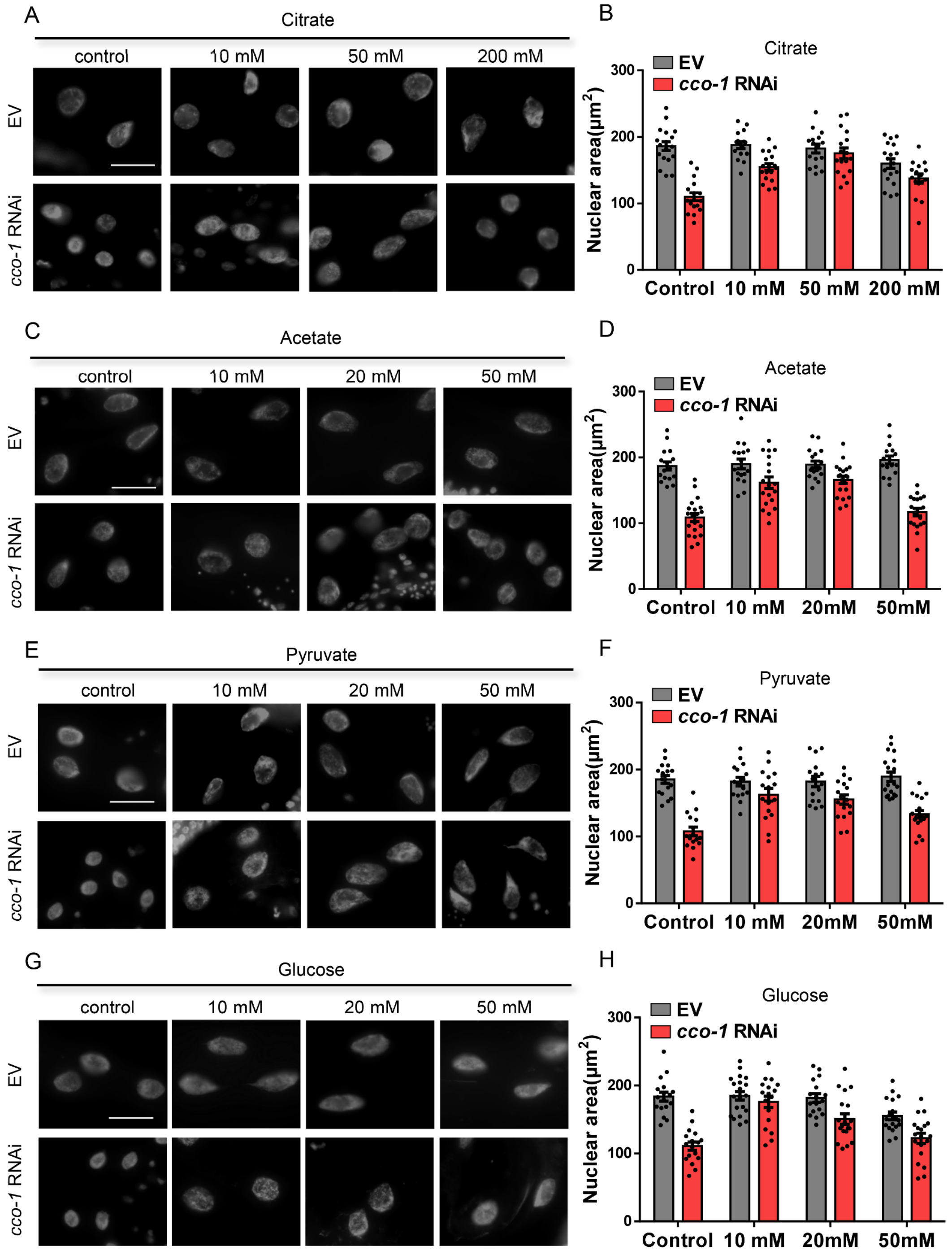

fig. S4. Adding substrates of acetyl-CoA during development attenuates chromatin reorganization under mitochondrial stress conditions. (**A**, **C**, **E**, and **G**) Representative images of DAPI immunostaining of intestinal nuclei in day 1 adult animals grown on EV or *cco-1* RNAi bacteria with or without the corresponding metabolites treatment during development. DAPI (grey); Scale bar, 25 μm. (**B**, **D**, **F**, and **H**) Quantification of intestinal nuclear maximum cross section area at day 1 of adulthood in animals as shown in (A, C, E, and G). n $\geqslant$ 15 nuclei.

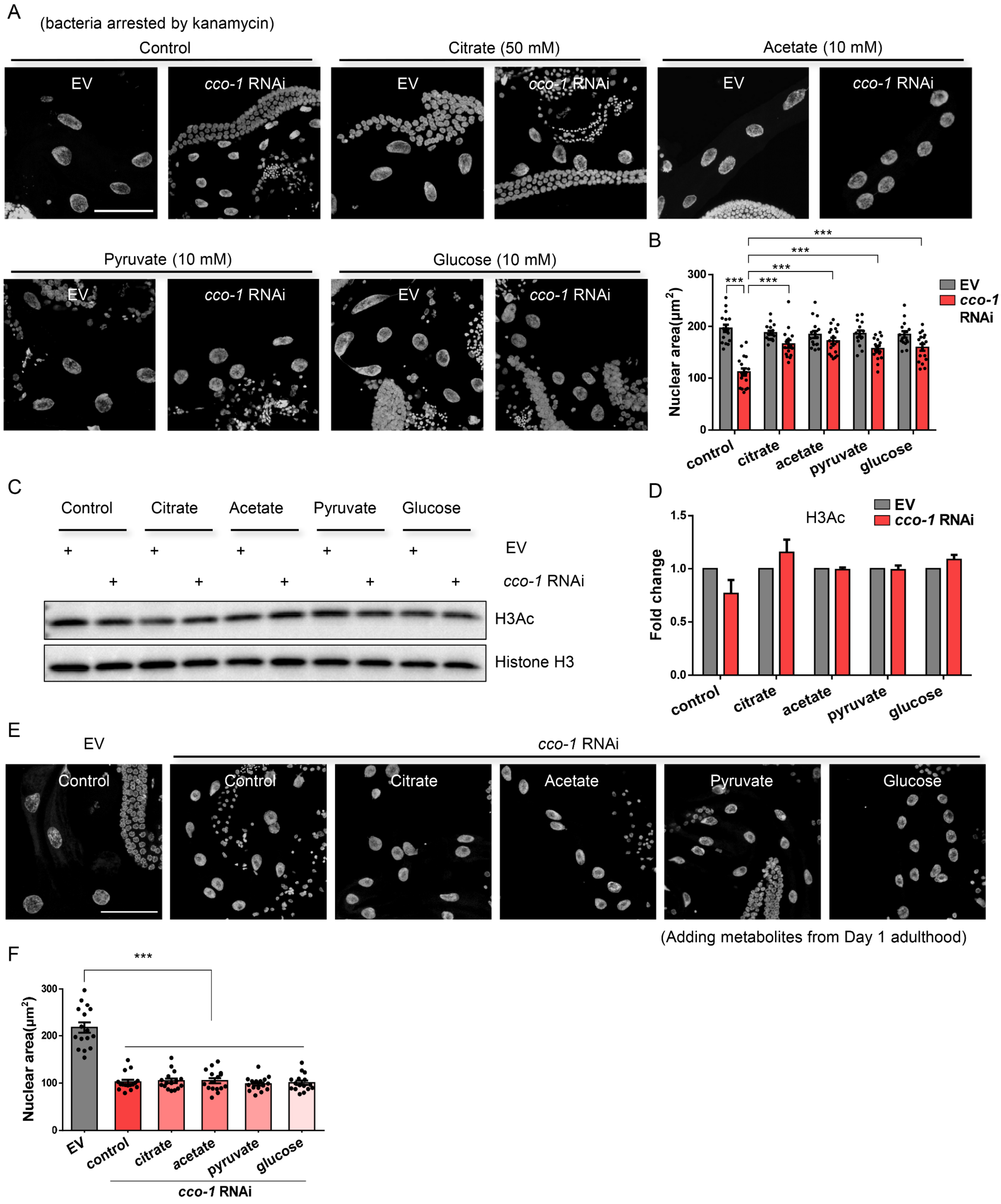

fig. S5. Adding substrates of acetyl-CoA during adulthood has no effect on chromatin structure under mitochondrial stress conditions. (**A**) Representative maximal intensity projection images of DAPI immunostaining of intestinal nuclei in day 1 adult animals grown on EV or *cco-1* RNAi bacteria arrested by kanamycin with or without metabolites treatment (as indicated). DAPI (grey). Scale bar,

50 µm. (**B**) Quantification of the intestinal nuclear maximum cross section area at day 1 of adulthood in animals as shown in (A). $n \geq 15$ nuclei. (**C**) Immunoblots of histone H3Ac in WT animals grown on EV or *cco-1* RNAi with or without citrate (50 mM), acetate (10 mM), pyruvate (10 mM), or glucose (10 mM). Anti-histone H3 serves as loading control. (**D**) Quantified mean data of histone H3Ac levels (relative to histone H3). $n = 2$. (**E**) Representative maximal intensity projection images of DAPI immunostaining of intestinal nuclei in day 3 adult animals grown on EV or *cco-1* RNAi bacteria from hatch with or without metabolites treatment (as indicated) only during adulthood. DAPI (grey); Scale bar, 50 µm. (**F**) Quantification of the intestinal nuclear maximum cross section area at day 3 of adulthood in animals as shown in (E). $n \geq 15$ nuclei.

***$p < 0.0001$ via t-test. Error bars, SEM.

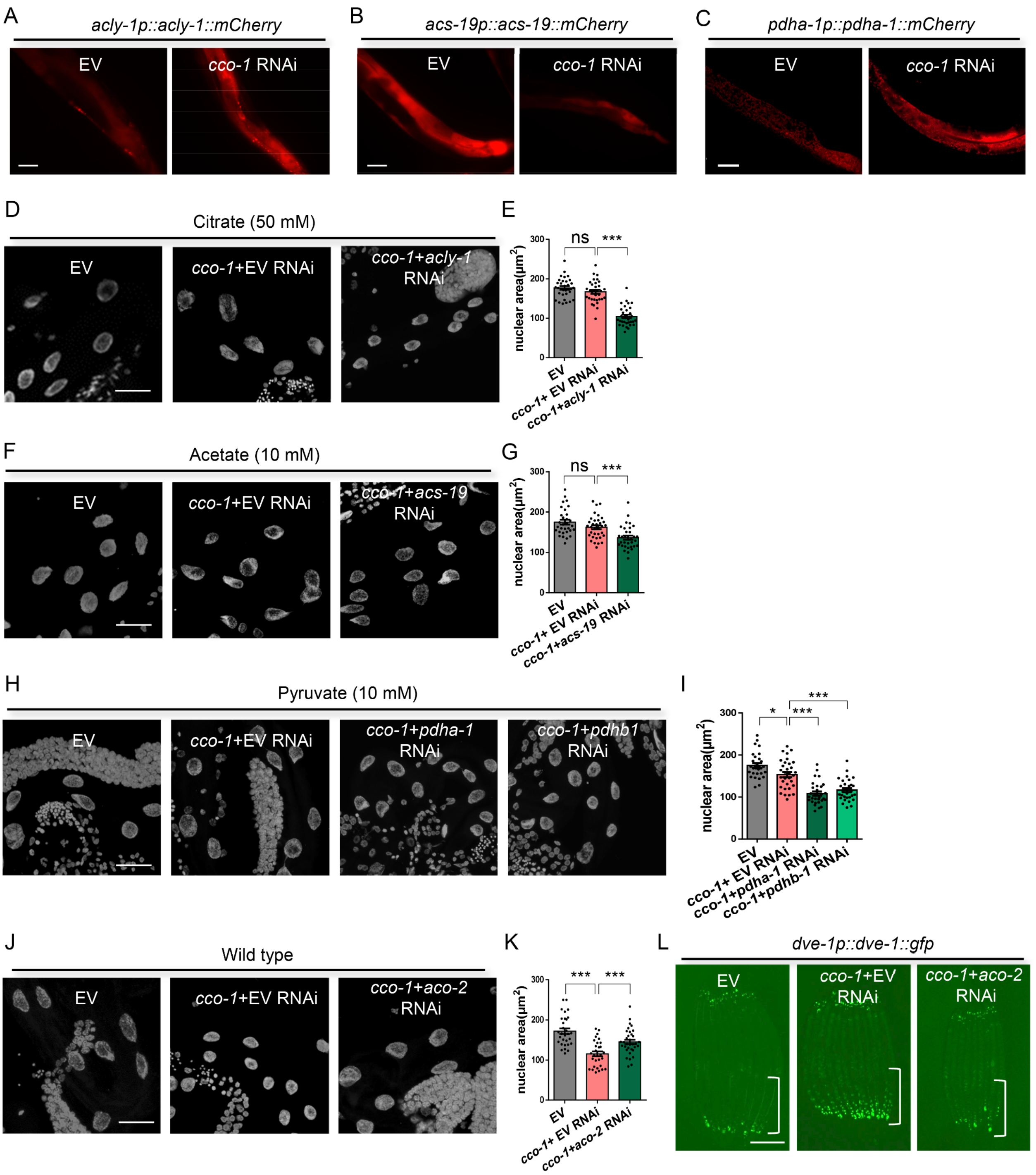

fig. S6. Enzymes involved in acetyl-CoA synthesis respond to mitochondrial stress. (**A**) Representative photomicrographs of *acly-1p::acly-1::mCherry* animals grown on EV or *cco-1* RNAi bacteria from hatch. Scale bar, 25 μm. (**B**) Representative photomicrographs of *acs-19p::acs-19::mCherry* animals grown on EV or *cco-1* RNAi bacteria from hatch. Scale bar, 25 μm. (**C**) Representative photomicrographs of *pdha-1p::pdha-1::mCherry* animals grown on EV or *cco-1* RNAi bacteria from hatch. Scale bar, 25 μm. (**D**) Representative maximal intensity projection images of

DAPI immunostaining of intestinal nuclei in day 1 adult animals grown on EV, *cco-1*+EV, or *cco-1+acly-1* double RNAi bacteria with 50 mM citrate treatment. DAPI (grey). Scale bar, 25 µm. (**E**) Quantification of the intestinal nuclear maximum cross section area at day 1 of adulthood in animals as shown in (D). (**F**) Representative maximal intensity projection images of DAPI immunostaining of intestinal nuclei in day 1 adult WT, animals grown on EV, *cco-1*+EV, or *cco-1+acs-19* double RNAi bacteria with 10 mM acetate treatment. DAPI (grey). Scale bar, 25 µm. (**G**) Quantification of the intestinal nuclear maximum cross section area at day 1 of adulthood in animals as shown in (F). (**H**) Representative maximal intensity projection images of DAPI immunostaining of intestinal nuclei in day 1 adult WT, animals grown on EV, *cco-1*+EV, *cco-1+pdha-1*, or *cco-1+pdhb-1* double RNAi bacteria with 10 mM pyruvate treatment. DAPI (grey). Scale bar, 25 µm. (**I**) Quantification of the intestinal nuclear maximum cross section area at day 1 of adulthood in animals as shown in (H). (**J**) Representative maximal intensity projection images of DAPI immunostaining of intestinal nuclei in day 1 adult animals grown on EV, *cco-1*+EV, or *cco-1+aco-2* double RNAi bacteria from hatch. DAPI (grey). Scale bar, 25 µm. (**K**) Quantification of the intestinal nuclear maximum cross section area at day 1 of adulthood in animals as shown in (J). (**L**) Representative photomicrographs of *dve-1p::dve-1::gfp* animals grown on EV, *cco-1*+EV, or *cco-1+aco-2* double RNAi bacteria from hatch. Scale bar, 250 µm.

*$p < 0.05$, ***$p < 0.0001$, ns denotes $p > 0.05$ via t-test. Error bars, SEM; $n \geq 25$ nuclei.

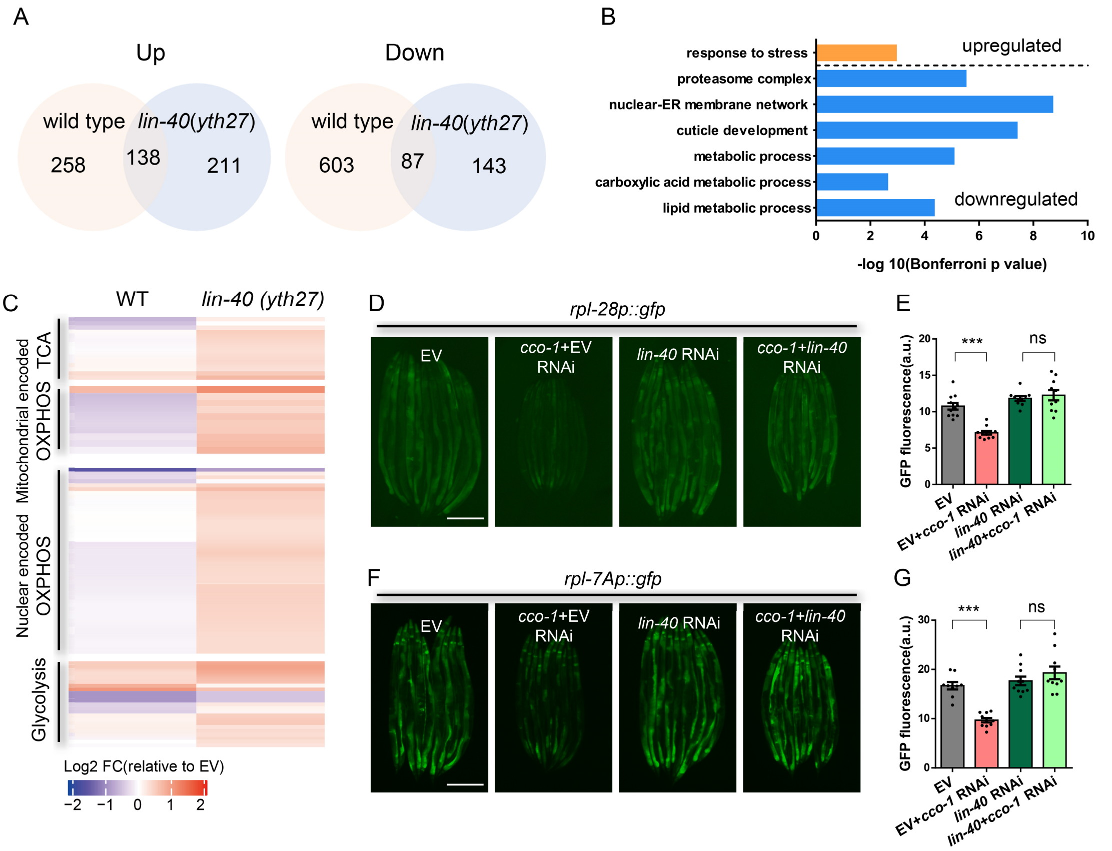

fig. S7. NuRD complex is required for the transcriptional response to the mitochondrial stress. (**A**) Venn diagram of differentially expressed genes (DEGs) in WT and *lin-40* mutant animals grown on *cco-1* RNAi bacteria for 2 days. Genes with adjusted p value < 0.05 were selected as DEGs. (**B**) Representative top GO terms of upregulated and downregulated genes dependent on *lin-40* (Bonferroni adjusted p value < 0.05). (**C**) Heat map of 33 TCA genes (cel00020), 111 OXPHOS genes (cel00190), and 42 glycolysis genes (cel00010) from the KEGG pathway database. Fold change (FC) was calculated by comparing normalized count values of each condition to each empty vector control and then transformed to log2 scale. Each condition was replicated three times. (**D**) Representative photomicrographs of *rpl-28p::gfp* animals grown on EV, *cco-1*+EV, EV+*lin-40*, or *cco-1*+*lin-40* double RNAi bacteria from hatch. Scale bar, 250 µm. (**E**) Quantification of *rpl-28p::gfp* expression of the entire intestine in animals as shown in (D). n ≥ 10 worms. (**F**) Representative photomicrographs of *rpl-7Ap::gfp* animals grown on EV, *cco-1*+EV, EV+*lin-40*, or *cco-1*+*lin-40* double RNAi bacteria from hatch. Scale bar, 250 µm. (**G**) Quantification of *rpl-7Ap::gfp* expression of the entire intestine in

animals as shown in (F). $n \geq 10$ worms.

***$p < 0.0001$, ns denotes $p > 0.05$ via t-test. Error bars, SEM.

See Data file S2 for list of genes differentially expressed with *cco-1* RNAi treatment.

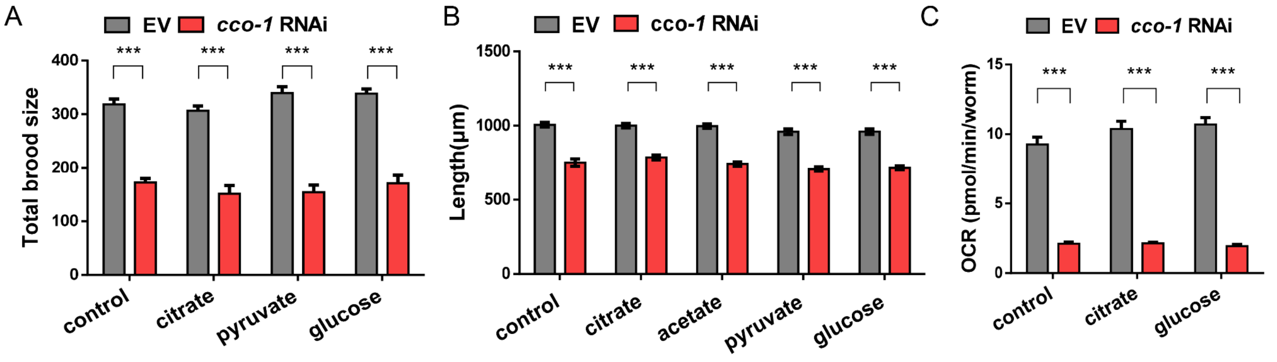

fig. S8. Adding substrates of acetyl-CoA does not restore the decreased oxygen consumption rate and fecundity under mitochondrial stress conditions. (**A**) Supplementation of citrate (50 mM), pyruvate (10 mM), or glucose (10 mM) does not restore *cco-1* RNAi-induced fecundity decrease. n = 6 worms. (**B**) Supplementation of citrate (50 mM), acetate (10 mM), pyruvate (10 mM), or glucose (10 mM) does not restore *cco-1* RNAi-triggered body length decrease. n = 15 worms. (**C**) Supplementation of citrate (50 mM) or glucose (10 mM) does not restore *cco-1* RNAi-induced oxygen consumption rate (OCR) decrease. n = 100 worms.

***p < 0.0001 via t-test. Error bars, SEM.

Table S1. Lifespan statistics (related to Fig. 6).

Related Figures	Repeat	Strains	Median lifespan (Days)	Total Animals	Censored Animals	P-value
**Fig. 6A**	1#	**WT; EV**	22	161	6	
		lin-40(*yth27*); EV	10	153	16	***< 0.0001
		lin-53(*n833*); EV	18	153	19	***< 0.0001
		**WT; *cco-1* RNAi**	30	163	12	
		lin-40(*yth27*)*; cco-1* RNAi	12	157	8	***< 0.0001
		lin-53(*n833*)*; cco-1* RNAi	18	153	11	***< 0.0001
	2#	**WT; EV**	24	143	13	
		lin-40(*yth27*); EV	10	123	21	***< 0.0001
		lin-53(*n833*); EV	20	148	12	***< 0.0001
		**WT; *cco-1* RNAi**	30	128	9	
		lin-40(*yth27*)*; cco-1* RNAi	12	126	23	***< 0.0001
		lin-53(*n833*)*; cco-1* RNAi	18	122	21	***< 0.0001
**Fig. 6C**	1#	**WT**	21	172	8	
		LIN-40 over-expression	25	173	7	***< 0.0001
	2#	**WT**	21	147	6	
		LIN-40 over-expression	25	154	19	***< 0.0001
**Fig. 6D**	1#	**WT; EV**	21	169	3	
		WT; EV; citrate	21	166	7	0.2684(ns)
		WT; EV; acetate	18	153	16	***< 0.0001
		**WT; *cco-1* RNAi**	32	160	15	
		WT; *cco-1* RNAi; citrate	26	168	7	***< 0.0001
		WT; *cco-1* RNAi; acetate	22	163	17	***< 0.0001
	2#	**WT; EV**	22	138	6	
		WT; EV; citrate	22	141	5	0.6443(ns)
		WT; EV; acetate	20	137	9	**< 0.001
		**WT; *cco-1* RNAi**	28	140	11	
		WT; *cco-1* RNAi; citrate	24	128	6	***< 0.0001
		WT; *cco-1* RNAi; acetate	22	139	8	***< 0.0001
**Fig. 6E**	1#	**WT; EV**	21	153	2	
		WT; EV; pyruvate	16	166	10	**< 0.001
		WT; EV; glucose	18	167	6	*< 0.05
		**WT; *cco-1* RNAi**	29	163	12	
		WT; *cco-1* RNAi; pyruvate	22	177	3	***< 0.0001
		WT; *cco-1* RNAi; glucose	18	185	10	***< 0.0001
	2#	**WT; EV**	22	134	5	
		WT; EV; pyruvate	19	144	11	**< 0.001

		WT; EV; glucose	18	144	9	**< 0.001
		**WT; *cco-1* RNAi**	29	141	8	
		WT; *cco-1* RNAi; pyruvate	23	132	13	***< 0.0001
		WT; *cco-1* RNAi; glucose	22	146	5	***< 0.0001

Table S2. List of *C. elegans* strains

Strains	Source	WormBase ID
Bristol (N2) strain as wild-type (WT)	CGC	N2
SJ4100 (zcIs13[*hsp-6p::gfp*] V)	CGC	SJ4100
SJ4005 (zcIs4[*hsp-4p::gfp*] V)	CGC	SJ4005
CL2070 (dvIs70[*hsp-16.2p::gfp* + *rol-6*(*su1006*)])	CGC	CL2070
SJ4197 (zcIs39[*dve-1p::dve-1::gfp*])	CGC	SJ4197
OH10993(otEx4944[*lin-53p::lin-53::gfp* + *rol-6*]	CGC	OH10993
MT10408(nEx998[*lin-53p::gfp* + *unc-76*(+)])	CGC	MT10408
MT14761(*lin-53*(*n833*) I)	CGC	MT14761
VC3201(*atfs-1*(*gk3094*) V)	CGC	VC3201
BC15433(*dpy-5*(*e907*) I; sEx15433[*rpl-28p::gfp* + *pCeh361*])	CGC	BC15433
BC15215(*dpy-5*(*e907*) I; sEx15215[*rpl-7Ap::gfp* + *pCeh361*])	CGC	BC15215
SJZ47(foxSi16[*myo-3p::tomm-20::mKate2::HA::tbb-2* 3' UTR] I)	CGC	SJZ47
SJZ204(foxSi37[*ges-1p::tomm-20::mKate2::HA::tbb-2* 3' UTR] I)	CGC	SJZ204
LTY747(ythIs27[*lin-40p::lin-40::FLAG::mCherry* + *rol-6*])	This study	N/A
LTY795(ythIs23[*lin-53p::lin-53::gfp* + *rol-6*])	This study	N/A
LTY561(ythIs16[*dve-1p::mCherry* + *rol-6*])	This study	N/A
LTY913(*lin-40*(*yth27*) V)	This study	N/A
LTY1284(ythEx187[*acly-1p::acly-1::mCherry* + *rol-6*])	This study	N/A
LTY1286(ythEx189[*acs-19p::acs-19:mCherry* + *rol-6*])	This study	N/A
LTY1416(ythEx202*[pdha-1p::pdha-1:mCherry + rol-6]*	This study	N/A
LTY797(ythIs27[*lin-40p::lin-40::Flag::mCherry* + *rol-6*]; *atfs-1*(*gk3094*) V)	This study	N/A
LTY844(zcIs39[*dve-1p::dve-1::gfp*] II; *lin-40*(*yth27*) V)	This study	N/A
LTY39(ythIs3[*rgef-1p::egl-20* + *myo-2p::tomato*] IV; zcIs39[*dve-1p::dve-1::gfp*] II)	Zhang et al., 2018	N/A
LTY1221(ythIs23[*lin-53p::lin-53::gfp* + *rol-6*]; *atfs-1*(*gk3094*) V)	This study	N/A
LTY451(*lin-40*(*yth27*) V; ythIs3[*rgef-1p::egl-20+myo-2p::tomato*] IV; zcIs39[*dve-1p::dve-1::gfp*] II)	This study	N/A
PHX1984 (syb1984[*dve-1::gfp*] X)	Sunybiotech	N/A
PHX2115 (syb2115[*lin-40::gfp*] V)	Sunybiotech	N/A
